# AI-Derived Blood Biomarkers for Ovarian Cancer Diagnosis: Systematic Review and Meta-Analysis

**DOI:** 10.2196/67922

**Published:** 2025-03-24

**Authors:** He-Li Xu, Xiao-Ying Li, Ming-Qian Jia, Qi-Peng Ma, Ying-Hua Zhang, Fang-Hua Liu, Ying Qin, Yu-Han Chen, Yu Li, Xi-Yang Chen, Yi-Lin Xu, Dong-Run Li, Dong-Dong Wang, Dong-Hui Huang, Qian Xiao, Yu-Hong Zhao, Song Gao, Xue Qin, Tao Tao, Ting-Ting Gong, Qi-Jun Wu

**Affiliations:** 1 Department of Clinical Epidemiology Shengjing Hospital of China Medical University ShenYang China; 2 Clinical Research Center Shengjing Hospital of China Medical University ShenYang China; 3 Liaoning Key Laboratory of Precision Medical Research on Major Chronic Disease Shengjing Hospital of China Medical University ShenYang China; 4 Department of Epidemiology School of Public Health China Medical University ShenYang China; 5 Department of Obstetrics and Gynecology Shengjing Hospital of China Medical University ShenYang China; 6 Department of Undergraduate Shengjing Hospital of China Medical University ShenYang China; 7 NHC Key Laboratory of Advanced Reproductive Medicine and Fertility (China Medical University) National Health Commission ShenYang China

**Keywords:** artificial intelligence, AI, blood biomarker, ovarian cancer, diagnosis, PRISMA

## Abstract

**Background:**

Emerging evidence underscores the potential application of artificial intelligence (AI) in discovering noninvasive blood biomarkers. However, the diagnostic value of AI-derived blood biomarkers for ovarian cancer (OC) remains inconsistent.

**Objective:**

We aimed to evaluate the research quality and the validity of AI-based blood biomarkers in OC diagnosis.

**Methods:**

A systematic search was performed in the MEDLINE, Embase, IEEE Xplore, PubMed, Web of Science, and the Cochrane Library databases. Studies examining the diagnostic accuracy of AI in discovering OC blood biomarkers were identified. The risk of bias was assessed using the Quality Assessment of Diagnostic Accuracy Studies–AI tool. Pooled sensitivity, specificity, and area under the curve (AUC) were estimated using a bivariate model for the diagnostic meta-analysis.

**Results:**

A total of 40 studies were ultimately included. Most (n=31, 78%) included studies were evaluated as low risk of bias. Overall, the pooled sensitivity, specificity, and AUC were 85% (95% CI 83%-87%), 91% (95% CI 90%-92%), and 0.95 (95% CI 0.92-0.96), respectively. For contingency tables with the highest accuracy, the pooled sensitivity, specificity, and AUC were 95% (95% CI 90%-97%), 97% (95% CI 95%-98%), and 0.99 (95% CI 0.98-1.00), respectively. Stratification by AI algorithms revealed higher sensitivity and specificity in studies using machine learning (sensitivity=85% and specificity=92%) compared to those using deep learning (sensitivity=77% and specificity=85%). In addition, studies using serum reported substantially higher sensitivity (94%) and specificity (96%) than those using plasma (sensitivity=83% and specificity=91%). Stratification by external validation demonstrated significantly higher specificity in studies with external validation (specificity=94%) compared to those without external validation (specificity=89%), while the reverse was observed for sensitivity (74% vs 90%). No publication bias was detected in this meta-analysis.

**Conclusions:**

AI algorithms demonstrate satisfactory performance in the diagnosis of OC using blood biomarkers and are anticipated to become an effective diagnostic modality in the future, potentially avoiding unnecessary surgeries. Future research is warranted to incorporate external validation into AI diagnostic models, as well as to prioritize the adoption of deep learning methodologies.

**Trial Registration:**

PROSPERO CRD42023481232; https://www.crd.york.ac.uk/PROSPERO/view/CRD42023481232

## Introduction

### Ovarian Cancer Diagnosis: Status and Demands

Ovarian cancer (OC) is the deadliest gynecologic malignancy, characterized by nonspecific symptoms that often remain undetected until the disease has progressed [1,2]. The conventional diagnosis of OC principally depends on imaging techniques (encompassing ultrasound, computed tomography, and magnetic resonance imaging); serum biomarkers (such as cancer antigen 125, carcinoembryonic antigen, and human epididymis protein 4); along with the invasive procedure (histological biopsy) [3,4]. However, the sensitivity and specificity of imaging techniques and biomarkers are restricted [5]. Furthermore, the histopathological test is inherently invasive [3]. Therefore, there is an urgent need for more accurate, noninvasive, and reliable diagnostic methods.

### Potential of Noninvasive Blood Markers for OC

Minimally invasive diagnostic procedures, particularly the use of blood samples, are among the foremost common methods of detection [6]. Moreover, patients are generally more willing to undergo blood tests, leading to higher compliance rates [7]. Blood contains a rich repertoire of biomolecules, including proteins, nucleic acids, and metabolites, which can potentially serve as indicators of OC diagnosis [8-11]. The development of omics has opened new doors for biomarker discovery. The genomics, proteomics, and metabolomics of blood samples can provide a wealth of information about the molecular changes in cancer [12]. For instance, Ke et al [13] systematically investigated OC metabolism through the metabolic profiling of 448 plasma samples. The analysis of dysregulated metabolic pathways extends our current understanding of OC metabolism. Similarly, Dhar et al [8] applied glycoproteomics to serum of women with OC or benign pelvic masses and healthy controls and analyzed glycosylation patterns in serum markers and supported the hypothesis that blood glycoproteomic profiling can be used for OC diagnosis and staging. Notably, the exponential growth of multiomics data has presented a major challenge that surpasses traditional analytic capabilities [14,15]. Fortunately, artificial intelligence (AI) algorithms offer a solution [16,17]. AI can manage complex datasets and spot hidden patterns and potential biomarkers, enabling more accurate OC diagnosis and personalized treatment.

### Application of AI in OC Blood Markers

AI, particularly machine learning (ML) and deep learning (DL), has attracted increasing attention in medical research due to its capability to analyze large biomedical datasets [18-23]. AI-driven models have emerged as a promising tool for developing predictive models for OC by analyzing complex and multidimensional datasets to uncover biomarkers. For instance, a multicenter retrospective study screened 52 features from laboratory tests in blood samples to build an ML model. Integrating 20 base AI models, it performed well internally and externally, outperforming the CA125 and HE4 biomarkers in identifying OC [24]. In addition, a blood-based metabolite panel demonstrated independent predictive ability and complemented the risk of ovarian malignancy algorithm for distinguishing early-stage OC from benign disease to better inform clinical decision-making [25]. Despite such encouraging results, the related results are scattered. Whether the application of AI in OC blood biomarker research can significantly improve diagnostic accuracy remains controversial.

### Purpose of This Study (AI and OC Blood Markers)

Therefore, there is a crucial need for a high-quality synthesis of the available evidence. The purpose of this study is to provide a systematic overview of the diagnostic accuracy of AI techniques in identifying OC blood markers as well as to elucidate their applicability, potential, and limitations.

## Methods

### Protocol Registration and Study Design

This meta-analysis was conducted in adherence to the PRISMA (Preferred Reporting Items for Systematic Reviews and Meta-Analyses) checklist (Multimedia Appendix 1) and MOOSE (Meta-Analysis of Observational Studies in Epidemiology) reporting guidelines [26,27]. The protocol was prospectively registered with the International Prospective Register of Systematic Reviews (PROSPERO; CRD42023481232).

### Literature Search and Eligibility Criteria

A comprehensive search of the MEDLINE, Embase, IEEE Xplore, PubMed, Web of Science, and the Cochrane Library databases was carried out from inception to January 16, 2024. Detailed search strategies are summarized in Multimedia Appendix 2. Two independent investigators (MQJ and HLX) assessed the records after removing the duplicates at the title and abstract level, and finally at the full-text level, according to the inclusion and exclusion criteria (Textbox 1). Two investigators (MQJ and XYL) independently appraised the articles for eligibility. An inconsistency in selection was reconciled through discussion with a third independent investigator (HLX).

**Inclusion and exclusion criteria.**

**Inclusion criteria**

- Population: adults (aged ≥18 years) with potential ovarian cancer lesions
- Intervention: artificial intelligence–assisted blood test
- Comparison: histopathology or other reliable clinical diagnosis
- Outcomes: diagnostic performance (ie, sensitivity and specificity or detailed information that could extract or construct 2×2 contingency tables)
- Studies: original articles (ie, observational studies or randomized controlled trials)
- Language: English

**Exclusion criteria**

- Population: nonhuman samples or other diseases
- Intervention: nonblood samples or no artificial intelligence algorithms
- Comparison: no control group
- Outcomes: no diagnostic performance data (ie, 2×2 contingency tables cannot be extracted or constructed from the provided data)
- Studies: records, such as letters, conference abstracts, case reports, or review articles
- Language: non-English

### Data Extraction

Data were independently extracted by 2 investigators (QPM and XYL) using a predefined data extraction sheet, with any discrepancies resolved through the adjudication of a third investigator (HLX). Necessary data of 2×2 contingency tables that included true positives (TP), false positives, true negatives (TN), and false negatives were extracted. The details of the data are presented in Multimedia Appendix 3 [8,24,25,28-64]. In cases where these values were not explicitly reported, values were calculated using descriptive statistics available in the study. For studies presenting multiple contingency tables, either for identical or disparate AI algorithms, each table was treated as an independent result [65].

### Study Quality Assessment

The risk of bias and concerns about the applicability of all included studies were assessed by 2 independent investigators (MQJ and HLX), using the Quality Assessment of Diagnostic Accuracy Studies–Artificial Intelligence (QUADAS-AI) criteria [66]. Conflicts were discussed and solved with a third investigator (XYL). The risk of bias assessment included 4 domains: patient selection, index test, reference standard, and flow and timing. For assessing clinical applicability, only the first 3 domains were evaluated [66,67]. In addition, the median was used as the threshold for determining the risk level of bias, with studies classified as low risk if ≥2 domains were deemed as low risk and high risk if <2 domains were considered low risk [68].

### Data Analysis

We used the bivariate diagnostic random effects model to compute the summary receiver operating characteristics to determine summary estimates of the sensitivity, specificity, and area under the curve (AUC) with their respective 95% CIs [69]. Sensitivity was defined as the probability of a person with OC having a positive test result, indicating the capacity of the index test to identify patients, considered by the equation: sensitivity = TP / (TP +  false negatives). Specificity was the probability of a woman without OC having a negative test result, reflecting the test's ability to correctly identify OC-free individuals calculated by the equation: specificity = TN / (false positives + TN) [70,71]. The performance of the test can also be assessed using the AUC. This area may be interpreted as the probability that a random woman with OC has a higher value of the measurement than a random person without OC. In general, an AUC of >0.80 is considered good [72]. A perfect test would have an AUC of 1 and a useless test would have an AUC of 0.5 [73].

Study heterogeneity was determined using the *I*^2^ statistic and the Q test [74]. When the same or different AI models were tested within the same article, the proposed model with the best accuracy was used for further meta-analysis [65]. Subgroup and regression analyses were performed to explore potential sources of heterogeneity. Sensitivity analysis was implemented via a qualitative systematic review of studies from which concatenated tables could not be extracted or constructed. Publication bias was assessed using funnel plot asymmetry test by Deeks et al [75].

Subgroup analyses were performed according to the following: (1) AI algorithms (ML or DL), (2) external validation (yes or no), (3) levels of risk of bias (low or high), (4) year of publication (after or before 2022), (5) geographical distribution (Asia, North America, or Europe), (6) sample size (>300 or ≤300 as median), (7) blood sample type (serum or plasma), (8) biomarkers type (protein or mixed), and (9) number of modeling biomarkers (>8 or ≤8 as median).

The variability in sensitivity and specificity estimates was graphically represented through a cross-hairs plot, generated using R software (version 4.2.1; R Foundation for Statistical Computing) [76]. All other statistical analyses were conducted in Stata (version 17.0; StataCorp). The statistical significance was defined as *P*<.05.

## Results

### Study Selection and Characteristics of Eligible Studies

**Figure 1:**
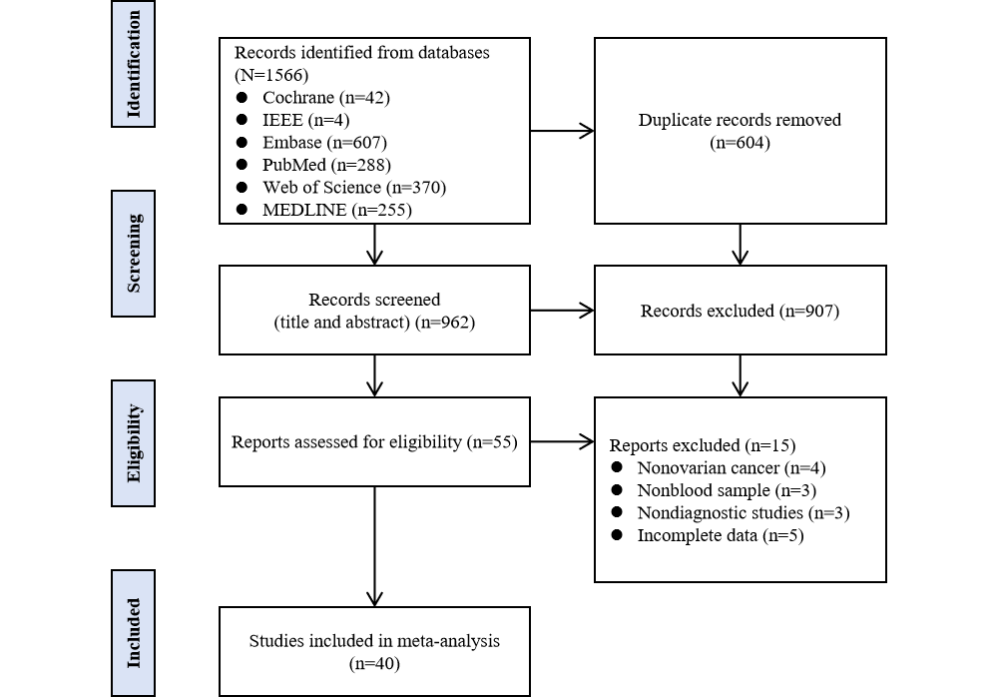
PRISMA (Preferred Reporting Items for Systematic Reviews and Meta-Analyses) flow diagram for study selection and processing in the meta-analysis.

The database search identified 1566 records from which 604 duplicates were removed. We then performed the title and abstract screening of 962 records and subsequently a full-text evaluation of 55 records. Following the exclusion of 15 articles, as detailed in Multimedia Appendix 4 [77-91], a total of 40 studies were included in this meta-analysis (Figure 1).

Most of the studies were performed with retrospectively (21/40, 52%) and prospectively (18/40, 45%) collected data, and one study collected both retrospective and prospective data (Table 1). In total, 12% (5/40) of studies sourced their data from public databases. In terms of AI algorithm types, a total of 36 (90%) studies were classified as ML, whereas 10% (4/40) of studies were classified as DL. Most (36/40, 90%) of the studies validated their algorithms, while only some (7/40, 18%) studies carried out an external validation (Table 2). The blood samples were mainly serum (27/40, 68%), and the type of blood biomarkers was mainly protein (25/40, 62%; Table 3).

**Table 1:** Baseline characteristics (study design, data source, selection criteria, time frame, age, and sample size) of 40 included studies on artificial intelligence–based ovarian cancer diagnosis with blood samples.

Study	Study design	Data source	Selection criteria	Time frame	Age (y), mean or median	Sample size, n
Cai et al [24], 2024	Retrospective	Data from Tongji Hospital, Tongji Medical College, Huazhong University of Science and Technology, central China; Women’s Hospital, School of Medicine, Zhejiang University, eastern China; and Sun Yat-Sen University Cancer Center, southern China	Patients with a history of other malignant cancers or precancers, pregnancy in the last 6 mo, or affected by HIV; not newly diagnosed in any of the 3 hospitals; individuals without any available laboratory tests were excluded	From January 2012 to April 2021	Cancer: (53/51/56)^a^; control: (34/34/48)^a^	10,992 (3007/5641/2344)^a^
Abuzinadah et al [28], 2023	Retrospective	Data from the Third Affiliated Hospital of Soochow University	All patients underwent postoperative case diagnosis, and none of them had received preoperative radiotherapy or chemotherapy	From July 2011 to July 2018	NR^b^	349 (244/105)^c^
Bifarin and Fernandez [29], 2024	Retrospective	Data from a serum lipidomic analysis of ovarian cancer patients of Korean descent	NR	NR	NR	325 (227/98)^c^
Cameron et al [30], 2023	Retrospective	Data from the Welcome Trust Clinical Research Facility at the Western General Hospital, Edinburgh, the Emergency Medicine Research Group at the Edinburgh Royal Infirmary, the Beatson West of Scotland Cancer Centre in Glasgow, the University of Swansea, Royal Preston Hospital, and Manchester Cancer Research Centre	NR	NR	Ovary: 61; NCS^d^ female individuals only: 56	385 (NR)
Chen et al [31], 2023	Retrospective	Data from the Department of Gynecology of Harbin Medical University Cancer Hospital, Gene Expression Omnibus database, UCSC Xena	Patients with a primary radiological diagnosis of ovarian tumor; newly diagnosed patients without any significant comorbidities or history of previous malignancies; willingness to participate in the study and provision of written informed consent	From December 2020 to July 2021	NR	44 (44)^e^
Dhar et al [8], 2023	Retrospective	Data from Indivumed (Hamburg, Germany)	NR	NR	NR	351 (237/114)^c^
Hamidi et al [32], 2023	Retrospective	Data from the Gene Expression Omnibus database, a Japanese nationwide research project, and patients with cancer who were referred or admitted to the National Cancer Center Hospital	The serum samples for noncancer controls who had no history of cancer and no hospitalization during the previous 3 months were collected; patients with cancer who were treated with preoperative chemotherapy and radiotherapy before serum collection were excluded	NR	Internal set: 52	3411 (2156/3079/92/240)^f^
Lai et al [33], 2023	Retrospective	Data from clinical laboratory examination	NR	From January 2013 to October 2022	46.4	778 (545/233)^g^
Li et al [34], 2024	Retrospective	Data from the Cancer Hospital of the University of Chinese Academy of Sciences (Zhejiang Cancer Hospital)	No patients with ovarian cancer received chemotherapy, radiotherapy, or surgery, no healthy donors had a history of cancer before sample collection	NR	Stage 1: 53.6; stage 2: 55.8; stage 3 and 4: 54.9; control: 34.1	69 (NR)
Reilly et al [35], 2023	Retrospective and prospective	Data from multiple studies spanning multiple centers	Patient age ≥18 years; informed consent provided by the patient to participate in research; patient agreeable to phlebotomy; patient had a documented adnexal mass	NR	47.5	2186 (NR)
Zhang et al [36], 2023	Retrospective	Data from patients who underwent physical examination at Chinese People’s Armed Police Force and First Medicine Center, People's Liberation Army General Hospital	Discharge diagnosis confirmed by clinical signs, imaging, and pathology for patients with gynecologic tumors and benign gynecologic diseases; if no histopathological examination was available, it was consistently confirmed by ≥2 types of imaging evidence, availability of laboratory test data at the time of first diagnosis, and blood collection before treatment	From January 2010 to June 2019	Ovarian cancer: (52.11/52.69/55.81)^h^; NOMGT^i^: (54.66/52.66/53.95)^h^; BGD^j^: (44.30/46.33/39.21)^h^; Healthy control: (49.96/47.26/49.67)^h^	1633(600/301)^g^
Ahamad et al [37], 2022	Retrospective	Data from Third Affiliated Hospital of Schow University	NR	From July 2017 to July 2018	NR	349 (85/21)^c^
Bahado-Singh et al [38], 2022	Prospective	Data from Oakland University William Beaumont School of Medicine	NR	NR	Cases: 66.2; control: 67.8	17 (NR)
Gupta et al [39], 2022	Retrospective	Data from 3 separate commercial biobanks: Dx Biosamples (San Diego, CA), Reprocell USA Inc (Beltsville, MD), and Fidelis Research AD (Sofa, Bulgaria)	NR	NR	NR	1243 (681)^k^
Hinestrosa et al [40], 2022	Retrospective	Data from a commercial biorepository (ProteoGenex, Inglewood, CA, United States)	The control group has no known history of cancer, autoimmune diseases, or neurodegenerative disorders, nor any presence of diabetes mellitus	From January 2014 to September 2020	60	323 (216/107)^c^
Irajizad et al [25], 2022	Prospective	Data from Anderson Cancer Center and at the Fred Hutchinson Cancer Research Center	NR	NR	NR	409 (108/118)^c^
Kim et al [41], 2022	Retrospective	Data from Oakland University William Beaumont School of Medicine	NR	NR	NR	269 (215)^e^
Li et al [42], 2022	Prospective	Data from 3 institutions	Women diagnosed with benign, borderline, and malignant ovarian tumors	From December 2018 to January 2020	NR	362 (178/184)^c^
Pais et al [43], 2022	Retrospective	Data from a commercially stored biological sample biobank (Invent diagnostica, Berlin, Germany)	NR	NR	NR	181 (NR)
Jeong et al [44], 2021	Retrospective	Data from Kangnam Sacred Heart Hospital	NR	From June 2014 to December 2020	Cancer: 54; control: 49	730 (511/219)^c^
Lu et al [45], 2020	Prospective	Data from the Third Affiliated Hospital of Soochow University	None of the patients with ovarian cancer received preoperative chemotherapy or radiotherapy	From July 2011 to July 2018	NR	349(235/114)^c^
Banaei et al [46], 2019	Prospective	Data from the UMass Memorial Medical Center Chemotherapy Infusion Center and Gastroenterology Clinics and Innovative Research	NR	NR	NR	20 (40/160)^c^
Whitwell et al [47], 2018	Retrospective	Data from a synthetic dataset modeled from the United Kingdom Collaborative Trial of Ovarian Cancer Screening	Trial participants at enrollment were postmenopausal women aged 50-74 y who had no family history of ovarian cancer	NR	NR	89 (NR)
Ivanova et al [48], 2016	Retrospective	Data from the clinical diagnostic laboratory of the LLC LYTECH, the Blokhin Cancer Research Center of Russian Academy of Medical Sciences, the National Research Center of Coloproctology, the Moscow Dermatovenerologic Dispensary, clinical hospitals of Peoples’ Friendship University of Russia	NR	NR	Cancer: 52; control: 49	67 (NR)
Jiang et al [49], 2013	Prospective	Data from the affiliated hospital, Sun Yat-Sen University	NR	NR	61.7	87 (51/36)^l^
Yang et al [50], 2013	Prospective	Data from the Peking University Third Hospital	NR	From January 2003 to December 2009	Stage I/II: 54.8; stage III: 57.3; stage IV: 58.2; normal: 52.8; carcinoid: 51.6	246 (NR)
Shan et al [51], 2012	Prospective	Data from the Tampa, Florida metropolitan area	Women with a prior unilateral or bilateral oophorectomy were ineligible, as were women with a previous history of cancer. All patients underwent preoperative radiologic imaging, either by pelvic ultrasound, CT, or MRI. Only patients who underwent surgery based on clinical suspicion of ovarian cancer were eligible	NR	NR	423 (NR)
Thakur et al [52], 2011	Prospective	Data from the FDA-NCI clinical proteomics program databank	NR	NR	NR	216 (173/43)^c^
Donach et al [53], 2010	Prospective	Data from Padua Hospital (now the Veneto Oncology Institute)	NR	From 1999 to 2005	48	201 (NR)
Ziganshin et al [54], 2008	Prospective	Data from Byelorussian Oncology Center with patients with ovarian cancer and the Clinical Diagnostic Laboratory with clinically healthy women	NR	NR	Cancer: 51;control: 49	118 (NR)
Liu et al [55], 2007	Prospective	Data from Northwestern University, Johns Hopkins University in Baltimore, MD, and the University of Innsbruck, Austria	Normal samples were from patients who had 4-year follow-up examinations to ensure that they did not have cancer at the time the samples were taken	From 1999 to 2002	NR	563 (315/78/170)^m^
Zhang et al [56], 2007	Prospective	Data from the Duke University Medical Center, Durham, NC, St Bartholomew’s Hospital, London, United Kingdom, and the Groningen University Hospital, Groningen, Netherlands	NR	NR	NR	468 (200/150)^c^
Chatterjee et al [57], 2006	Retrospective	Data from the Barbara Ann Karmanos Cancer Institute, the MD Anderson Cancer Center, Weill Medical College of Cornell University, Northwestern University Robert H Lurie Comprehensive Cancer Center, and the Gynecologic Oncology Group Tissue Bank	NR	NR	NR	129 (85/44)^c^
Lin et al [58], 2006	Prospective	Data from the Tri-Service General Hospital, Taiwan, and Republic of China	Patients with any history of cancer, operations that had removed body organ, or current chronic or acute diseases were excluded	NR	NR	65 (65)^e^
Liu [59], 2006	Prospective	Data from Clinical Proteomic Program Databank	NR	NR	NR	253 (NR)
Wu et al [60], 2006	Prospective	Data from the Tri-Service General Hospital, Taiwan	No history of gynecologic tumors and had a normal pelvic examination and pelvic sonography	NR	NR	65 (NR)
Li and Ramamohanrao [61],2004	Prospective	Data from a public website	NR	From November 2003	NR	253 (215/112)^c^
Li et al [62], 2004	Retrospective	Data from a public website	NR	From February 2002	NR	469 (100/116)^c^
Zhang et al [63], 1999	Retrospective	Data from an existing data set of clinically diagnosed with pelvic masses and University of Texas MD Anderson Cancer Center	NR	NR	NR	625 (174/255)^c^
Wilding et al [64], 1994	Prospective	Data from the Hospital of the University of Pennsylvania	Patients with carcinoma in situ were excluded	NR	NR	98 (NR)

^a^Training/external validation 1/external validation 2.

^b^NR: not reported.

^c^Training/testing.

^d^NCS: noncancer symptomatic.

^e^Training.

^f^Training/internal validation/external validation 1/external validation 2.

^g^Training/internal validation.

^h^Training/internal validation/external validation.

^i^NOMGT: nonovarian malignant gynecologic tumor.

^j^BGD: benign gynecologic disease.

^k^Testing.

^l^Training/prediction.

^m^Training/testing/internal validation.

**Table 2:** Artificial intelligence algorithm features (reference standard, algorithm type, type of internal validation, and external validation) of 40 included studies on artificial intelligence–based ovarian cancer diagnosis with blood samples.

Study	Reference standard	Algorithms	ML^a^ or DL^b^	Type of internal validation	External validation
Cai et al [24], 2024	Histopathology	MCF^c^, XGB^d^, LGBM^e^, CatBoost, GBM^f^, RF^g^, NB^h^, LR^i^	ML	5-fold cross-validation	Yes
Abuzinadah et al [28], 2023	Histopathology	RF, KNN^j^, SGD^k^, ETC^l^, XGB, GBM	ML	K-fold cross-validation	No
Bifarin and Fernandez [29], 2024	Histopathology	AutoML^m^, RF, SVM^n^, KNN	ML	5-fold cross-validation	No
Cameron et al [30], 2023	Histopathology	NR	ML	A nested cross-validation	No
Chen et al [31], 2023	Histopathology	CBS^o^, GISTIC^p^	ML	3-fold cross-validation	Yes
Dhar et al [8], 2023	Histopathology	LR	ML	10-fold cross-validation	No
Hamidi et al [32], 2023	NR	LR, DT^q^, RF, ANN^r^, XGB	ML	5-fold cross-validation	Yes
Lai et al [33], 2023	Histopathology	SVM	ML	A validation (unclear)	No
Li et al [34], 2024	Histopathology	LDA^s^, RF, NN^t^, SVM	ML	A validation (unclear)	No
Reilly et al [35], 2023	Histopathology	MIA3G^u^	DL	NR^v^	No
Zhang et al [36], 2023	Histopathology	LR, FLD^w^, SVM, RF, ANN	ML	Cross-validation	Yes
Ahamad et al [37], 2022	Histopathology	RF, SVM, DT, XGBM, LR, GBM, LGBM	ML	5-fold cross-validation	No
Bahado-Singh et al [38], 2022	NR	RF, SVM, LDA, PAM^x^, GLM^y^, DL	ML	10-fold cross-validation	No
Gupta et al [39], 2022	NR	OVR^z^	ML	NR	No
Hinestrosa et al [40], 2022	Histopathology	RFE^aa^	ML	5-fold cross-validation	No
Irajizad et al [25], 2022	NR	DL, RF, EL^ab^, GBM	DL	5-fold cross-validation	Yes
Kim et al [41], 2022	Histopathology	DT, LR, ANN, RF, SVM	ML	10-fold cross-validation	Yes
Li et al [42], 2022	Histopathology	SVM	ML	5-fold cross-validation	No
Pais et al [43], 2022	Histopathology	EvA-3^ac^, OSC^ad^	ML	A validation (unclear)	No
Jeong et al [44], 2021	NR	ROMA^ae^	ML	3-fold cross-validation	No
Lu et al [45], 2020	Histopathology	ROMA, DT, LR	ML	10-fold cross-validation	No
Banaei et al [46], 2019	NR	CT^af^, KNN	ML	5-fold cross-validation	No
Whitwell et al [47], 2018	Histopathology	Parenclitic networks, LR, RDLG^ag^	ML	Monte Carlo cross-validation	No
Ivanova et al [48], 2016	Histopathology	GA^ah^, SNN^ai^	ML	Leave one out cross-validations	No
Jiang et al [49], 2013	NR	ANN, CT, Split-point score analysis	ML	One cross-validation	No
Yang et al [50], 2013	Histopathology	ANN	ML	Blind test validation	No
Shan et al [51], 2012	Histopathology	HH-SVM^aj^	ML	5-fold cross-validation	No
Thakur et al [52], 2011	NR	ANNs, LDA	ML	Cross-validation	No
Donach et al [53], 2010	NR	ANN	ML	NR	No
Ziganshin et al [54], 2008	NR	GA, SNN	ML	Cross-validation	No
Liu et al [55], 2007	NR	PLS^ak^, SVM, DT C5.0	ML	10-fold cross-validation	No
Zhang et al [56], 2007	Histopathology	ANN	ML	Cross-validation	Yes
Chatterjee et al [57], 2006	NR	Feed-forward NN	DL	NR	No
Lin et al [58], 2006	NR	DT	ML	Cross-validation	No
Liu [59], 2006	NR	SVM	ML	10-fold cross-validation	No
Wu et al [60], 2006	Histopathology	CT	ML	Cross-validation	No
Li and Ramamohanrao [61], 2004	NR	SVM, NB, KNN, DT, CS4^al^	ML	10-fold cross-validation	No
Li et al [62], 2004	NR	SVM	ML	One out cross-validation	No
Zhang et al [63], 1999	Histopathology	ANN	ML	Cross-validation	No
Wilding et al [64], 1994	Histopathology	Backpropagation NN	DL	A validation (unclear)	No

^a^ML: machine learning.

^b^DL: deep learning.

^c^MCF: multi-criteria decision making-based classification fusion.

^d^XGB: extreme gradient boosting.

^e^LGBM: light gradient boosting machine.

^f^GBM: gradient boosting machine.

^g^RF: random forest.

^h^NB: naive Bayes.

^i^LR: logistic regression.

^j^KNN: k-nearest neighbor.

^k^SGD: stochastic gradient descent.

^l^ETC: extra-trees classifier.

^m^AutoML: automated machine learning.

^n^SVM: support vector machine.

^o^CBS: circular binary segmentation algorithm.

^p^GISTIC: the genomic identification of significant targets in cancer 2.0 algorithm.

^q^DT: decision tree.

^r^ANN: artificial neural network.

^s^LDA: Linear Discriminant Analysis.

^t^NN: neural network.

^u^MIA3G: multivariate index assay 3G.

^v^NR: not reported.

^w^FLD: Fisher linear discriminant.

^x^PAM: prediction analysis for microarrays.

^y^GLM: generalized linear model.

^z^OVR: *One Versus Rest* classifier multiclass classification model.

^aa^RFE: Recursive Feature Elimination.

^ab^EL: ensemble learning.

^ac^Eva-3: evolutionary algorithm 3

^ad^OSC: compose the classification algorithm.

^ae^ROMA: risk of ovarian malignancy algorithm.

^af^CT: classification tree.

^ag^RDLG: raw data logistic regression.

^ah^GA: genetic algorithm.

^ai^SNN: supervised neural network.

^aj^HH-SVM: hybrid huberized support vector machine.

^ak^PLS: partial least-square regression.

^al^CS4: cascading-and-sharing for ensembles of decision trees.

**Table 3:** Biomarker characteristics (blood sample type, detection method, biomarker type, number of modeling, and detection biomarkers) of 40 included studies on artificial intelligence–based ovarian cancer diagnosis with blood samples.

Study	Blood sample type	Device or method	Biomarker type	Number of modeling biomarkers	Number of detection marker
Cai et al [24], 2024	Blood	NR^a^	Protein, mixed	52	NR
Abuzinadah et al [28], 2023	Blood	General chemical tests, blood routine tests	Mixed	49	49
Bifarin and Fernandez [29], 2024	Serum	NR	Mixed	17	17
Cameron et al [30], 2023	Serum	Spectrum	Mixed	5	5
Chen et al [31], 2023	Plasma	LC-WGS^b^	DNA	NR	NR
Dhar et al [8], 2023	Serum	LCMS^c^	Protein	27	571
Hamidi et al [32], 2023	Serum	miRNA labeling kit and miRNA Oligo Chip	RNA	10	2568
Lai et al [33], 2023	Serum	MS^d^	Protein	7	95
Li et al [34], 2024	Plasma	Nanoflow cytometry, SEC^e^, CA125 ELISA^f^ kit, and HE4 ELISA kit	Protein	7	7
Reilly et al [35], 2023	Serum	Roche cobas 6000 clinical analyzer	Protein	7	7
Zhang et al [36], 2023	Blood	NR	Mixed	25	25
Ahamad et al [37], 2022	Blood and serum	NR	Mixed	47	47
Bahado-Singh et al [38], 2022	Plasma	Illumina Infnium MethylationEPIC BeadChip arrays or methylation analysis	DNA	25	179,238
Gupta et al [39], 2022	Serum	UHPLC‑MS^g^	Mixed	25	6336
Hinestrosa et al [40], 2022	Plasma	ACE^h^	Protein	34	42
Irajizad et al [25], 2022	Plasma	LCMS analysis	Mixed	7	475
Kim et al [41], 2022	Serum	Immunoassay, C8000 analyzer, diazo reagent	Mixed	NR	NR
Li et al [42], 2022	Plasma	QIAamp Circulating Nucleic Acid kit	DNA	5	1272
Pais et al [43], 2022	Serum	MALDINR-TOF MS^i^	Protein	CHCA^j^:26-57; SA^k^:12-113	CHCA:8500; SA:8500
Jeong et al [44], 2021	Serum	2-step chemiluminescent microparticle immunoassay	Protein	16	3
Lu et al [45], 2020	Serum	Roche Cobas 8000 modular analyzer series	Protein	2	2
Banaei et al [46], 2019	Serum	A microfluidic SERS^l^-based immunoassay method	Protein	5	5
Whitwell et al [47], 2018	Serum	Olink’s multiplex immunoassay Oncology II panel	Protein	92	92
Ivanova et al [48], 2016	Serum	MALDI-TOF MS	Protein	7	200-400
Jiang et al [49], 2013	Serum	ELISA	Protein	5	174
Yang et al [50], 2013	Serum	SELDI-TOF MS^m^	Protein	184	184
Shan et al [51], 2012	Serum	Liquid chromatography electrospray tandem mass spectrometry	Mixed	18	18
Thakur et al [52], 2011	Serum	SELDI-TOF MS	Protein	NR	NR
Donach et al [53], 2010	Serum	Radioimmunoassay kits	Protein	4	6
Ziganshin et al [54], 2008	Serum	MALDI-TOF MS or Ultraflex TOF mass spectrometer	Protein	MB-IMAC Cu^n^:13; MB-WCX:11	MB-HIC8^o^:135;MB-HIC18^p^:137; MB-IMAC Cu:115; MB-WCX^q^:96
Liu et al [55], 2007	Serum	prOTOF MS	Protein	NR	96
Zhang et al [56], 2007	Serum	Radioimmunoassay kits	Protein	4	4
Chatterjee et al [57], 2006	Serum	ELISA	Protein	65	65
Lin et al [58], 2006	Plasma	SELDI analysis,WCX2 chip analysis,SAX2 chip analysis	Protein	3	4
Liu [59], 2006	Serum	MS	Protein	NR	NR
Wu et al [60], 2006	Plasma	SELDI-TOF MS	Protein	5	NR
Li and Ramamohanrao [61], 2004	Serum	MS	Protein	72	15,154
Li et al [62], 2004	Serum	SELDI-TOF MS	Protein	10	15,155
Zhang et al [63], 1999	Serum	Radioimmunoassay kits, a spectrophotometric method with a kit	Mixed	4	4
Wilding et al [64], 1994	Serum and plasma	Radioimmunoassay	Mixed	8	8

^a^NR: not reported.

^b^LC-WGS: low-coverage whole genome sequencing.

^c^LC-MS: liquid chromatography-mass spectrometry.

^d^MS: mass spectrometry.

^e^SEC: size exclusion chromatography.

^f^ELISA: enzyme-linked immunosorbent assay.

^g^UHPLC-MS: ultra-high performance liquid chromatography-mass spectrometry

^h^ACE: alternating current electrokinetics.

^i^MALDINR-TOF MS: matrix-assisted laser desorption/Ionization neutral reflector time-of-flight mass spectrometry.

^j^CHCA: α-Cyano-4-hydroxycinnamic acid matrix.

^k^SA: sinapinic acid matrix.

^l^SERS: surface-enhanced Raman spectroscopy.

^m^SELDI -TOF MS: surface-enhanced laser desorption and ionization mass spectrometry.

^n^MB-IMAC Cu: magnetic beads MB-IMAC Cu.

^o^MB-HIC8: magnetic beads MB-HIC 8.

^p^MB-HIC18: magnetic beads MB-HIC 18.

^q^MB-WCX: magnetic beads MB-WCX.

### Quality Assessment

The quality of the included studies was appraised using the QUADAS-AI (Figures S1 and S2 in Multimedia Appendix 5). In detail, most of the studies were rated as having a high or unclear risk of bias based on patient selection (22/40, 55%) and index test (33/40, 82%) domains. These assessments might be attributed to the absence of explicit delineation of included patients, such as previous testing history and clinical setting, as well as to deficiencies in rigorous external validation of the AI models.

### Pooled Performance of AI Algorithms

**Figure 2:**
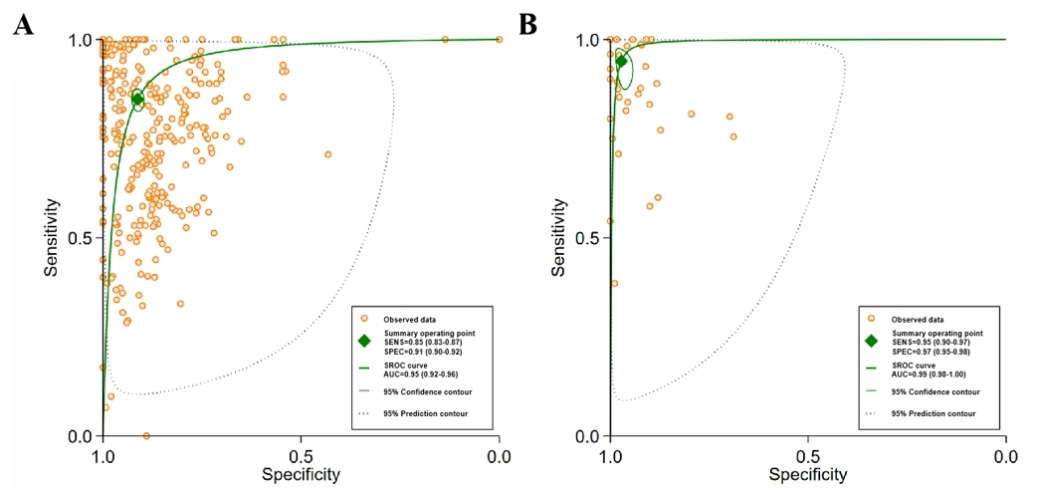
Summary receiver operating characteristic (SROC) curves of all studies included in the meta-analysis (n=40). (A) SROC curves of all studies included in the meta-analysis (40 studies with 342 tables). (B) SROC curves of studies when selecting contingency tables reporting the highest accuracy (40 studies with 40 tables). AUC: area under the curve; SENS: summary sensitivity; SPEC: summary specificity.

**Figure 3:**
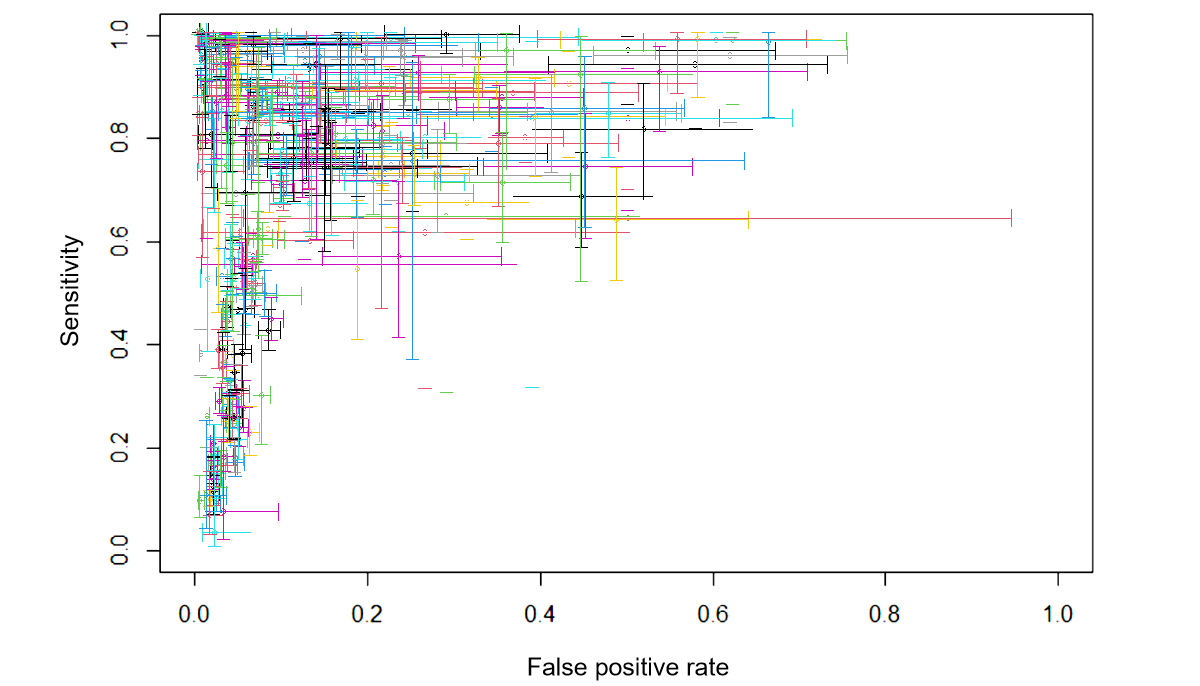
Cross-hair plot of all studies included in the meta-analysis (n=40).

The summary receiver operating characteristics curves for the 40 included studies with 342 contingency tables are shown in Figure 2. The pooled sensitivity and specificity were 85% (95% CI 83%-87%) and 91% (95% CI 90%-92%), respectively, with an AUC of 0.95 (95 % CI 0.92-0.96) for all AI algorithms. Notably, when contingency tables with the highest accuracy were extracted from each study, the pooled sensitivity and specificity were 95% (95% CI 90%-97%) and 97% (95% CI 95%-98%), respectively, with an AUC of 0.99 (95% CI 0.98-1.00). Reported point estimates and CIs of all included studies are shown in a cross-hairs plot (Figure 3).

### Subgroup Analyses and Meta-Regression Analysis

Results of the subgroup analyses revealed that acceptable diagnostic performance was observed in all subgroups, ranging from 74% to 98% for sensitivity and 85% to 96% for specificity. Detailed results are shown in Table 4, and the corresponding plots are presented in Figures S3-S21 in Multimedia Appendix 5. We divided the studies into subgroups according to the modalities of algorithms (Figures S3 and S13 in Multimedia Appendix 5), existence of external validation (Figures S4 and S14 in Multimedia Appendix 5), levels of risk of bias (Figures S5 and S15 in Multimedia Appendix 5), year of publication (Figures S6 and S16 in Multimedia Appendix 5), geographical distribution (Figures S7 and S17 in Multimedia Appendix 5), sample size (Figures S8 and S18 in Multimedia Appendix 5), blood sample type (Figures S9 and S19 in Multimedia Appendix 5), biomarkers type (Figures S10 and S20 in Multimedia Appendix 5), and number of modeling biomarkers (Figures S11 and S21 in Multimedia Appendix 5).

**Table 4:** Summary estimate of pooled performance of artificial intelligence–derived blood biomarkers for the diagnosis of ovarian cancer.

		Number of studies, n (%)	Sensitivity				*P* value^a^		Specificity				*P* value^a^
			Sensitivity (95% CI)	*I*^2^（95% CI）	*P* value^b^			Specificity (95% CI)		*I*^*2*^（95% CI)	*P* value^b^		
Overall		40 (100)	0.85 (0.83-0.87)	97.54 (97.41-97.66)	<.001	—^c^		0.91 (0.90-0.92)		99.15 (99.12-99.18)	<.001	—	
**Artificial intelligence algorithms^d^**							.07						.10
	Machine learning	36 (90)	0.86 (0.83-0.88)	97.79 (97.67-97.90)	<.001	—		0.92 (0.90-0.93)		99.27 (99.24-99.30)	<.001	—	
	Deep learning	4 (10)	0.77 (0.70-0.82)	85.81 (81.19-90.42)	<.001	—		0.85 (0.83-0.87)		68.55 (55.84-81.26)	<.001	—	
**External validation**							<.001						<.001
	Yes	7 (18)	0.74 (0.69-0.79)	98.35 (98.23-98.46)	<.001	—		0.94 (0.93-0.95)		99.54 (99.52-99.57)	<.001	—	
	No	33 (82)	0.90 (0.88-0.92)	94.91 (94.48-95.34)	<.001	—		0.89 (0.86-0.91)		94.17 (93.65-94.68)	<.001	—	
**Levels of risk of bias^e^**							<.001						.28
	Low	31 (78)	0.81 (0.79-0.84)	97.40 (97.25-97.55)	<.001	—		0.91 (0.89-0.92)		99.07 (99.03-99.11)	<.001	—	
	High	9 (22)	0.98 (0.96-0.99)	95.51 (94.74-96.27)	<.001	—		0.94 (0.89-0.96)		96.85 (96.37-97.33)	<.001	—	
**Year of publication**							<.001						<.001
	After 2022	19 (48)	0.81 (0.78-0.83)	97.44 (97.28-97.59)	<.001	—		0.90 (0.88-0.91)		99.04 (98.99-99.08)	<.001	—	
	Before 2022	21 (52)	0.95 (0.91-0.97)	96.83 (96.47-97.19)	<.001	—		0.96 (0.93-0.97)		97.64 (97.39-97.88)	<.001	—	
**Geographical distribution**							0.01						.92
	Asia	18 (45)	0.82 (0.78-0.84)	97.78 (97.65-97.91)	<.001	—		0.91 (0.89-0.92)		99.23 (99.20-99.26)	<.001	—	
	North America	15 (38)	0.92 (0.88-0.95)	94.47 (93.70-95.24)	<.001	—		0.91 (0.87-0.93)		95.88 (95.35-96.40)	<.001	—	
	Europe	7 (18)	0.91 (0.81-0.96)	96.54 (95.69-97.38)	<.001	—		0.96 (0.90-0.98)		97.02 (96.33-97.71)	<.001	—	
**Sample size**							<.001						.06
	>300	21 (52)	0.83 (0.80-0.85)	97.78 (97.65-97.91)	<.001	—		0.91 (0.89-0.92)		99.24 (99.21-99.27)	<.001	—	
	≤300	19 (48)	0.92 (0.88-0.95)	93.93 (93.07-94.79)	<.001	—		0.94 (0.91-0.96)		92.88 (91.82-93.94)	<.001	—	
**Blood sample type^f^**							<.02						.12
	Serum	27 (68)	0.94 (0.92-0.96)	98.13 (97.98-98.29)	<.001	—		0.96 (0.95-0.98)		98.57 (98.46-98.68)	<.001	—	
	Plasma	8 (20)	0.83 (0.78-0.87)	83.32 (79.03-87.61)	<.001	—		0.91 (0.88-0.94)		70.15 (61.13-79.17)	<.001	—	
**Marker type^g^**							.12						<.001
	Protein	25 (62)	0.87 (0.82-0.91)	98.65 (98.56-98.75)	<.001	—		0.95 (0.93-0.96)		99.65 (99.63-99.66)	<.001	—	
	Mixed	12 (30)	0.79 (0.77-0.82)	94.74 (94.27-95.21)	<.001	—		0.86 (0.84-0.88)		98.56 (98.47-98.64)	<.001	—	
**Number of modeling marker^h^**							.54						.31
	>8	15 (38)	0.82 (0.79-0.85)	97.73 (97.59-97.87)	<.001	—		0.90 (0.88-0.92)		99.20 (99.16-99.23)	<.001	—	
	≤8	14 (35)	0.79 (0.74-0.83)	88.11 (85.74-90.49)	<.001	—		0.90 (0.88-0.92)		79.83 (75.13-84.52)	<.001	—	

^a^*P* value for heterogeneity between subgroups with meta-regression analysis.

^b^*P* value for heterogeneity within each subgroup.

^c^Not applicable.

^d^Artificial intelligence algorithms include machine learning and deep learning.

^e^Low: ≥2 domain low risk; high: <2 domain low risk.

^f^Five articles that used incomplete information on blood sample type were excluded from this subgroup analysis.

^g^Three DNA studies that used DNA and 1 study that used RNA were excluded for this subgroup analyses.

^h^In total, 11 articles used incomplete information on the number of model markers were excluded for this subgroup analysis.

The meta-analysis uncovered substantial heterogeneity among studies, as evidenced by an *I*^2^ of 97.54% (*P*<.05) for sensitivity and 99.15% (*P*<.05) for specificity (Figure S12 in Multimedia Appendix 5). To further explore the causes of study heterogeneity, a meta-regression analysis was conducted (Table 4). The results showed that both external validation and year of publication were significant factors that influenced study heterogeneity with regard to sensitivity and specificity. Subgroups based on levels of risk of bias, geographical distribution, sample size, and blood sample type showed intergroup heterogeneity in the sensitivity of prediction (*P*<.05). In terms of marker type, specificity presented significant heterogeneity between groups (*P*<.05).

### Sensitivity Analyses and Publication Bias

A qualitative systematic review was performed for studies lacking directly or indirectly available contingency tables. The findings from this review were in alignment with the main analysis (Tables S1-S3 in Multimedia Appendix 6 [77-81]). In addition, the analysis did not reveal any publication bias in this meta-analysis (*P*=.72; Multimedia Appendix 7).

## Discussion

### Principal Findings

The burgeoning evolution of AI within the medical field has captured the attention of an increasing cadre of researchers, particularly in its applicability to disease diagnosis [92,93]. To the best of our knowledge, this meta-analysis is a pioneering effort specifically exploring the efficacy of AI in OC diagnosis via blood biomarkers. AI algorithms exhibited exceptional diagnostic capabilities for OC, boasting a pooled sensitivity of 85% (95% CI 83%-87%) and specificity of 91% (95% CI 90%-92%). Moreover, we identified substantial heterogeneity among the selected studies and determined the potential contributing factors through subgroup and meta-regression analyses. Overall, these results should be interpreted with caution as described by the constraints mentioned in subsequent sections.

### Heterogeneity

Heterogeneity is an inevitable problem in meta-analyses [94]. Significant interstudy heterogeneity was noted in terms of sensitivity (*I*^2^=97.54%) and specificity (*I*^2^=99.15%) in this study. External validation emerged as a crucial variable influencing study heterogeneity. Studies without external validation might yield results that were hard to generalize owing to factors such as sample selection bias and the characteristics of the research setting [95]. To address this, future research should focus on standardizing and applying the validation procedures, thus getting closer to the goal of providing more accurate and reliable diagnostic tools for clinical practice [96]. Besides, several factors contributed to the heterogeneity observed in sensitivity in this study. Studies with a pronounced risk of bias were predisposed to introduce uncertainties. Such biases could stem from flaws in study design, improper data collection, or inappropriate statistical analyses, all of which might distort the true relationship between the biomarker and the condition under investigation [67,97]. On the other hand, geographic disparities might be attributed to a complex interplay of genetic polymorphisms and environmental factors that differentially modulate biomarker expression levels, which could result in significant variability in biomarker sensitivity [98,99]. In addition, larger sample sizes generally offer enhanced statistical power and precision, enabling more reliable estimations of biomarker performance [100]. Understanding and accounting for these factors comprehensively will help to further reduce heterogeneity and enhance the validity and clinical relevance of meta-analysis results, ultimately leading to more precise and useful diagnostic tools for clinical application.

### Implication of Blood Sample Types

Blood specimens are relatively stable and can be easily accessed. Therefore, blood-based biomarkers have been regarded as a minimally invasive method with great value for disease diagnosis [101,102]. Plasma and serum are rich sources of information regarding an individual’s health state and are the focus of this study’s investigation [103]. Particularly noteworthy is that serum samples showed higher sensitivity than plasma ones in this study, which can potentially be ascribed to multiple factors. First, the distinct sample preparation procedures for serum and plasma may lead to variations in the concentration and availability of biomarkers. Serum is obtained after blood clotting, during which certain intracellular components can be released, potentially augmenting the repertoire of biomarkers [104]. In contrast, the anticoagulants used in plasma collection may impede the integrity or accessibility of biomarkers [105]. Second, the microenvironment within serum and plasma varies. Serum contains a more intricate milieu of proteins, enzymes, and other biomolecules that can interact with cancer biomarkers in a manner that heightens their detectability [102]. For example, the presence of specific binding proteins or proteases in serum may modify the conformation of biomarkers, rendering them more amenable to detection by the analytic methods used [106]. Furthermore, the centrifugation processes involved in separating serum and plasma can differentially partition the biomarkers. The speed, duration, and temperature of centrifugation may cause certain biomarkers to be preferentially retained in the serum fraction, contributing to the observed higher sensitivity [107]. In addition, the limited number of included studies for plasma may contribute to this phenomenon to some extent. Therefore, it is of paramount importance to judiciously select the sample type in the context of developing and implementing blood-based biomarker assays for OC.

### Implication of Algorithm Types

In subgroup analysis, ML surpassed DL in both sensitivity and specificity. This phenomenon warrants an in-depth exploration of the underlying reasons and improvement directions. The edge of ML likely stems from its algorithmic traits. For structured and well-defined data, traditional algorithms (eg, logistic regression and support vector machine) can adeptly capture biomarker-disease associations via mathematical and statistical tenets, yielding high diagnostic accuracy [108,109]. DL, empowered by its strong automatic feature extraction and complex architecture, can theoretically handle large data and extract deep patterns [110]. However, in this study, the number of studies included for DL was only 4, compared with 36 for ML, presumably constraining the exertion of DL’s advantages. The scant number of DL studies gives rise to data that are circumscribed in both sample variety and the expanse of feature distribution. To break through this dilemma, several optimization strategies can be considered. First, data augmentation, such as random rotations, scaling, flipping, and noise addition to the data can enhance dataset diversity, facilitating the DL model to learn more extensive features and patterns for better generalization [110-112]. Second, transfer learning is applicable. Using pretrained models from related medical or bioanalysis fields and fine-tuning with OC data, the DL model can draw on prior knowledge, accelerating training convergence and potentially improving performance [113,114]. In addition, model compression techniques, including pruning (ie, eliminating less important connections or neurons to maintain performance while reducing complexity) and quantization (ie, lowering parameter precision for faster inference and less memory use) can be used [115]. While these strategies hold promise, their implementation and efficacy in the context of OC diagnosis warrant further investigation and optimization.

### Implication of External Validation

At present, the data amassed for AI applications in OC diagnosis is circumscribed by the paucity of diverse external validation. Many studies rely on a single dataset for discovery, with cross-validation to estimate algorithm performance. Given the generalizable issues to unseen data, accuracy drops when tested on other research datasets, and substantially when tested on clinical data [116,117]. To address these challenges, several strategies can be implemented. First, multicenter collaborations should be actively pursued. Combining data from different medical institutions and regions to build a heterogeneous and comprehensive dataset and exposing algorithms to wider patient characteristics and biomarker profiles will enhance generalizability [118-120]. Second, standardized data collection and annotation protocols are crucial. They ensure data consistency and comparability among studies, minimizing variability from inconsistent methods [121]. This allows algorithms to be trained on more reliable and reproducible data, strengthening the foundation for AI application. Moreover, continuous evaluation and improvement of algorithms in clinical settings are essential. Prospective studies integrating the algorithms into routine practice and monitoring their performance can offer valuable feedback [122]. This iterative testing and refinement process helps algorithms adapt to clinical complexity, leading to more accurate OC diagnostic tools. Despite persisting challenges, we anticipate that these efforts will incrementally enhance diagnostic accuracy. Sustained refinement and collaboration are essential to exploiting the full potential of AI in OC diagnosis.

### Future Directions

Beyond the previous mentions, the existing literature in the field exhibits certain areas of improvement to reduce the gap between research and deployment. First, one of the richest data sources of patient health and clinical history is embedded in the electronic health records of a patient but remains hugely underutilized. AI’s ability to integrate blood biomarkers with other clinically relevant nonblood biomarkers, such as age, cancer history, and family history of cancer, could potentially outpace current practices if trained on sufficiently extensive datasets [123]. Future research could explore the synergistic integration of AI tools with clinical expertise, echoing a more realistic clinical scenario. Second, the problem of explainability is the subject of intensive research and various initiatives. Although symbolic AI or simple ML models, such as decision trees or linear regression, are still fully understood by people, understanding becomes increasingly difficult with more advanced techniques and is now impossible with many DL models; this situation can lead to unexpected results and nondeterministic behavior [124,125]. Third, data privacy and patient consent are critical concerns that need to be addressed before adopting the use of AI in clinical practice [126]. The integration of AI into clinical workflows requires careful consideration of ethical, legal, and regulatory aspects [127,128]. Transparent guidelines and regulations should be established to govern the use of AI in health care and ensure its responsible and ethical implementation.

### Strengths and Limitations

Our meta-analysis has several strengths. First, to the best of our knowledge, it represents a novel effort as the first systematic review and meta-analysis dedicated to evaluating the diagnostic performance of blood biomarker-based AI for OC. Our findings illuminate the considerable potential AI holds in this domain, while also highlighting the advantages of blood tests, such as their noninvasive nature, better patient compliance, and cost-effectiveness. Second, our comprehensive investigation included multiple subgroup analyses, all of which yielded acceptable diagnostic performance for the AI model. Third, the stringent quality assessment of all included studies was conducted using QUADAS-AI tool. In addition, the robustness of our meta-analysis was reinforced through a sensitivity analysis underpinned by a qualitative systematic review.

The results of our meta-analysis are likely to be overestimated or underestimated for some reasons. One limitation lies in the high heterogeneity of the studies included. Nonetheless, we thoroughly explored potential sources of between-study heterogeneity through meta-regression and subgroup analyses. Another limitation is that the contingency tables of 5 studies included in our systematic review were not directly or indirectly available. These studies provided only indicators, such as AUC, accuracy, and *F*_1_-score, which did not allow for the construction of contingency tables [77-81]. Nevertheless, we conducted a qualitative systematic review of these 5 studies and discovered that the findings aligned with the main analysis. Moreover, although no publication bias was noticed, it is still highly likely that there is unpublished material for this topic from the ever-growing nature of the framework and the likelihood of undisclosed research for commercial development [129]. Moreover, available AI research tends to be skewed toward the publication of positive results, indicating a potential publication bias.

### Conclusions

The findings of this study indicated that the use of AI for the analysis of noninvasive blood biomarkers in OC diagnostics holds substantial potential for achieving satisfactory predictive outcomes. Among the analyzed studies, those that used DL were notably fewer in number than those that used ML. This underscores a critical need for future research to prioritize the incorporation of DL methodologies. Furthermore, pursuing external validation datasets was a necessary avenue to optimize the performance and applicability of AI in this field.

## Appendix

Multimedia Appendix 1 PRISMA checklist.

Multimedia Appendix 2 Search terms and search strategy.

Multimedia Appendix 3 Contingency tables extracted from included studies.

Multimedia Appendix 4 The list of the excluded records during the process of full-text review.

Multimedia Appendix 5 The results of Quality Assessment of Diagnostic Accuracy Studies–Artificial Intelligence and subgroup analyses.

Multimedia Appendix 6 The characteristics for the 5 studies without sufficient data.

Multimedia Appendix 7 Publication bias.

## Abbreviations

AI: artificial intelligence
AUC: area under the curve
DL: deep learning
ML: machine learning
MOOSE: Meta-Analysis of Observational Studies in Epidemiology
OC: ovarian cancer
PRISMA: Preferred Reporting Items for Systematic Reviews and Meta-Analyses
QUADAS-AI: Quality Assessment of Diagnostic Accuracy Studies–Artificial Intelligence
TN: true negatives
TP: true positives

## Data Availability

The datasets generated or analyzed during this study are available from the corresponding author on reasonable request.
