# Supplementary material for: AI-Derived Blood Biomarkers for Ovarian Cancer Diagnosis: Systematic Review and Meta-Analysis
Source: J Med Internet Res. 2025 Mar 24;27:e67922. doi: 10.2196/67922 (PMC11976184; doi:10.2196/67922)
Supplement: Multimedia Appendix 2 [file jmir_v27i1e67922_app2.docx]

**[Multimedia Appendix](https://pmc.ncbi.nlm.nih.gov/articles/PMC10007007/#app1) 2. Search terms and search strategy**

**Pubmed**

("*Artificial intelligence*" OR "*machine learning*" OR "*deep learning* " OR "*neural network*") AND ("ovarian" OR "ovary") AND ("carcinoma" OR "tumor" OR "cancer" OR "neoplas*" ) AND ("blood " OR " plasma " OR " serum " OR "biomarker*" OR " marker*")

**Web of Science**

("Artificial intelligence" OR "machine learning" OR "deep learning" OR "neural network" ) AND ("ovarian" OR "ovary") AND ("carcinoma" OR "tumor" OR "cancer" OR "neoplas*" ) AND ("blood " OR " plasma " OR " serum " OR "biomarker* " OR " marker*")

**Cochrane**

#1 (Artificial intelligence) OR (machine learning) OR (deep learning) OR (neural network )

#2 (ovarian) OR (ovary)

#3 (carcinoma) OR (tumor) OR (cancer) OR (neoplas*)

#4 (blood) OR (plasma) OR (serum) OR (biomarker*) OR (marker*)

#5 #1 AND #2 AND #3 AND #4

**Embase**

('artificial intelligence' OR 'machine learning' OR 'deep learning' OR 'neural network') AND ('ovarian' OR 'ovary') AND ('carcinoma' OR 'tumor' OR 'cancer' OR 'neoplas*') AND ('blood' OR 'plasma' OR 'serum' OR 'biomarker*' OR 'marker*')

#1 'artificial intelligence' OR 'machine learning' OR 'deep learning' OR 'neural network'

#2 'ovarian' OR 'ovary'

#3 'carcinoma' OR 'tumor' OR 'cancer' OR 'neoplas*'

#4 'blood' OR 'plasma' OR 'serum' OR 'biomarker*' OR 'marker*'

#5 #1 AND #2 AND #3 AND #4

**IEEE**

("All Metadata":"artificial intelligence" OR "All Metadata":"machine learning" OR "All Metadata":"deep learning" OR "All Metadata":"neural network") AND ("All Metadata":"ovarian" OR "All Metadata":"ovary") AND ("All Metadata":"cancer" OR "All Metadata":"tumor" OR "All Metadata":"neoplas*" OR "All Metadata":"carcinoma") AND ("All Metadata":" blood" OR "All Metadata":" plasma" OR "All Metadata":"serum" OR "All Metadata":" biomarker*" OR "All Metadata":" marker*")

**Medline**

TX ( "*Artificial intelligence*" OR "*machine learning*" OR "*deep learning* " OR "*neural network*" ) AND TX ( "ovarian" OR "ovary" ) AND TX ( "carcinoma" OR "tumor" OR "cancer" OR "neoplas*" ) AND TX ( "blood " OR " plasma " OR " serum " OR "biomarker*" OR " marker*" )
