# Supplementary material for: AI-Derived Blood Biomarkers for Ovarian Cancer Diagnosis: Systematic Review and Meta-Analysis
Source: J Med Internet Res. 2025 Mar 24;27:e67922. doi: 10.2196/67922 (PMC11976184; doi:10.2196/67922)
Supplement: Multimedia Appendix 3 [file jmir_v27i1e67922_app3.docx]

**Multimedia Appendix 3. Contingency tables extracted from included studies ( 342 tables from 40 studies)**

| **Author [ref]** | **year** | **Total** | **TP** | **FP** | **FN** | **TN** | **SE** | **SP** | **AUC** |
| --- | --- | --- | --- | --- | --- | --- | --- | --- | --- |
| Cai et al. [24] | 2024 | 3007 | 560 | 92 | 153 | 2202 | 0.79 | 0.96 | 0.95 |
|  |  | 5641 | 496 | 248 | 217 | 2046 | 0.70 | 0.89 | 0.88 |
|  |  | 2344 | 555 | 369 | 158 | 1925 | 0.78 | 0.84 | 0.88 |
|  |  | 2739 | 405 | 73 | 40 | 2221 | 0.91 | 0.97 | 0.99 |
|  |  | 2495 | 111 | 87 | 90 | 2207 | 0.55 | 0.96 | 0.88 |
|  |  | 2885 | 511 | 115 | 80 | 2179 | 0.86 | 0.95 | 0.97 |
|  |  | 2385 | 35 | 25 | 56 | 2269 | 0.39 | 0.99 | 0.86 |
|  |  | 5372 | 176 | 290 | 22 | 4884 | 0.89 | 0.94 | 0.97 |
|  |  | 5370 | 109 | 621 | 87 | 4553 | 0.56 | 0.88 | 0.82 |
|  |  | 5543 | 249 | 398 | 120 | 4776 | 0.68 | 0.92 | 0.90 |
|  |  | 5272 | 39 | 114 | 59 | 5060 | 0.40 | 0.98 | 0.84 |
|  |  | 2170 | 165 | 148 | 54 | 1803 | 0.76 | 0.92 | 0.94 |
|  |  | 2112 | 99 | 289 | 62 | 1662 | 0.62 | 0.85 | 0.81 |
|  |  | 2293 | 245 | 203 | 97 | 1748 | 0.72 | 0.90 | 0.91 |
|  |  | 2000 | 14 | 117 | 35 | 1834 | 0.29 | 0.94 | 0.74 |
|  |  | 3007 | 544 | 67 | 169 | 2227 | 0.76 | 0.97 | 0.94 |
|  |  | 3007 | 547 | 83 | 166 | 2211 | 0.77 | 0.96 | 0.94 |
|  |  | 3007 | 549 | 67 | 164 | 2227 | 0.77 | 0.97 | 0.95 |
|  |  | 3007 | 544 | 71 | 169 | 2223 | 0.76 | 0.97 | 0.94 |
|  |  | 3007 | 484 | 64 | 229 | 2230 | 0.68 | 0.97 | 0.93 |
|  |  | 3007 | 537 | 330 | 176 | 1964 | 0.75 | 0.86 | 0.88 |
|  |  | 3007 | 523 | 108 | 190 | 2186 | 0.73 | 0.95 | 0.93 |
|  |  | 5641 | 291 | 331 | 176 | 4843 | 0.62 | 0.94 | 0.87 |
|  |  | 5641 | 293 | 373 | 174 | 4801 | 0.63 | 0.93 | 0.87 |
|  |  | 5641 | 294 | 347 | 173 | 4827 | 0.63 | 0.93 | 0.87 |
|  |  | 5641 | 302 | 373 | 165 | 4801 | 0.65 | 0.93 | 0.87 |
|  |  | 5641 | 290 | 341 | 177 | 4833 | 0.62 | 0.93 | 0.89 |
|  |  | 5641 | 353 | 885 | 114 | 4289 | 0.76 | 0.83 | 0.87 |
|  |  | 5641 | 322 | 517 | 145 | 4657 | 0.69 | 0.90 | 0.88 |
|  |  | 2344 | 270 | 256 | 123 | 1695 | 0.69 | 0.87 | 0.87 |
|  |  | 2344 | 273 | 279 | 120 | 1672 | 0.70 | 0.86 | 0.87 |
|  |  | 2344 | 280 | 287 | 113 | 1664 | 0.71 | 0.85 | 0.87 |
|  |  | 2344 | 273 | 248 | 120 | 1703 | 0.70 | 0.87 | 0.87 |
|  |  | 2344 | 273 | 209 | 120 | 1742 | 0.70 | 0.89 | 0.87 |
|  |  | 2344 | 332 | 552 | 61 | 1399 | 0.85 | 0.72 | 0.83 |
|  |  | 2344 | 280 | 269 | 113 | 1682 | 0.71 | 0.86 | 0.86 |
|  |  | 2495 | 108 | 73 | 93 | 2221 | 0.54 | 0.97 | 0.88 |
|  |  | 2495 | 107 | 78 | 94 | 2216 | 0.53 | 0.97 | 0.88 |
|  |  | 2495 | 108 | 71 | 93 | 2223 | 0.54 | 0.97 | 0.88 |
|  |  | 2495 | 107 | 83 | 94 | 2211 | 0.53 | 0.96 | 0.88 |
|  |  | 2495 | 81 | 57 | 120 | 2237 | 0.40 | 0.98 | 0.85 |
|  |  | 2495 | 100 | 330 | 101 | 1964 | 0.50 | 0.86 | 0.79 |
|  |  | 2495 | 93 | 108 | 108 | 2186 | 0.46 | 0.95 | 0.85 |
|  |  | 5370 | 96 | 440 | 100 | 4734 | 0.49 | 0.92 | 0.81 |
|  |  | 5370 | 104 | 481 | 92 | 4693 | 0.53 | 0.91 | 0.81 |
|  |  | 5370 | 99 | 460 | 97 | 4714 | 0.51 | 0.91 | 0.81 |
|  |  | 5370 | 100 | 455 | 96 | 4719 | 0.51 | 0.91 | 0.81 |
|  |  | 5370 | 104 | 409 | 92 | 4765 | 0.53 | 0.92 | 0.84 |
|  |  | 5370 | 136 | 1294 | 60 | 3881 | 0.69 | 0.75 | 0.80 |
|  |  | 5370 | 121 | 611 | 75 | 4563 | 0.62 | 0.88 | 0.83 |
|  |  | 2112 | 85 | 248 | 76 | 1703 | 0.53 | 0.87 | 0.79 |
|  |  | 2112 | 88 | 263 | 73 | 1688 | 0.55 | 0.87 | 0.79 |
|  |  | 2112 | 86 | 271 | 75 | 1680 | 0.54 | 0.86 | 0.79 |
|  |  | 2112 | 94 | 244 | 67 | 1707 | 0.59 | 0.88 | 0.80 |
|  |  | 2112 | 92 | 242 | 69 | 1709 | 0.57 | 0.88 | 0.80 |
|  |  | 2112 | 115 | 537 | 46 | 1414 | 0.71 | 0.73 | 0.75 |
|  |  | 2112 | 86 | 254 | 75 | 1697 | 0.54 | 0.87 | 0.78 |
|  |  | 3007 | 483 | 218 | 230 | 2076 | 0.68 | 0.91 | 0.89 |
|  |  | 3007 | 481 | 227 | 232 | 2067 | 0.68 | 0.90 | 0.88 |
|  |  | 3007 | 533 | 80 | 180 | 2214 | 0.75 | 0.97 | 0.94 |
|  |  | 3007 | 508 | 89 | 205 | 2205 | 0.71 | 0.96 | 0.93 |
|  |  | 3007 | 487 | 204 | 226 | 2090 | 0.68 | 0.91 | 0.89 |
|  |  | 3007 | 51 | 18 | 662 | 2276 | 0.07 | 0.99 | 0.65 |
|  |  | 5641 | 279 | 693 | 188 | 4481 | 0.60 | 0.87 | 0.78 |
|  |  | 5641 | 254 | 492 | 213 | 4682 | 0.54 | 0.91 | 0.78 |
|  |  | 5641 | 328 | 611 | 139 | 4563 | 0.70 | 0.88 | 0.88 |
|  |  | 5641 | 306 | 735 | 161 | 4439 | 0.66 | 0.86 | 0.84 |
|  |  | 5641 | 284 | 590 | 183 | 4584 | 0.61 | 0.89 | 0.80 |
|  |  | 5641 | 174 | 228 | 293 | 4946 | 0.37 | 0.96 | 0.81 |
|  |  | 2344 | 237 | 297 | 156 | 1654 | 0.60 | 0.85 | 0.80 |
|  |  | 2344 | 247 | 338 | 146 | 1613 | 0.63 | 0.83 | 0.80 |
|  |  | 2344 | 289 | 228 | 104 | 1723 | 0.74 | 0.88 | 0.89 |
|  |  | 2344 | 259 | 168 | 134 | 1783 | 0.66 | 0.91 | 0.87 |
|  |  | 2344 | 248 | 254 | 145 | 1697 | 0.63 | 0.87 | 0.82 |
|  |  | 2344 | 191 | 72 | 202 | 1879 | 0.49 | 0.96 | 0.86 |
|  |  | 2495 | 82 | 220 | 119 | 2074 | 0.41 | 0.90 | 0.77 |
|  |  | 2495 | 66 | 229 | 135 | 2065 | 0.33 | 0.90 | 0.75 |
|  |  | 2495 | 74 | 76 | 127 | 2218 | 0.37 | 0.97 | 0.85 |
|  |  | 2495 | 69 | 80 | 132 | 2214 | 0.34 | 0.97 | 0.84 |
|  |  | 2495 | 88 | 206 | 113 | 2088 | 0.44 | 0.91 | 0.78 |
|  |  | 2495 | 20 | 48 | 181 | 2246 | 0.10 | 0.98 | 0.63 |
|  |  | 5370 | 103 | 1087 | 93 | 4087 | 0.53 | 0.79 | 0.69 |
|  |  | 5370 | 79 | 585 | 117 | 4589 | 0.40 | 0.89 | 0.70 |
|  |  | 5370 | 114 | 735 | 82 | 4439 | 0.58 | 0.86 | 0.81 |
|  |  | 5370 | 115 | 843 | 81 | 4331 | 0.59 | 0.84 | 0.75 |
|  |  | 5370 | 111 | 999 | 85 | 4175 | 0.57 | 0.81 | 0.72 |
|  |  | 5370 | 57 | 326 | 139 | 4848 | 0.29 | 0.94 | 0.74 |
|  |  | 2112 | 93 | 466 | 68 | 1485 | 0.58 | 0.76 | 0.70 |
|  |  | 2112 | 78 | 293 | 83 | 1658 | 0.48 | 0.85 | 0.71 |
|  |  | 2112 | 97 | 226 | 64 | 1725 | 0.60 | 0.88 | 0.81 |
|  |  | 2112 | 98 | 302 | 63 | 1649 | 0.61 | 0.85 | 0.81 |
|  |  | 2112 | 92 | 425 | 69 | 1526 | 0.57 | 0.78 | 0.72 |
|  |  | 2112 | 58 | 96 | 103 | 1855 | 0.36 | 0.95 | 0.77 |
|  |  | 3007 | 538 | 115 | 175 | 2179 | 0.76 | 0.95 | 0.94 |
|  |  | 5641 | 321 | 569 | 146 | 4605 | 0.69 | 0.89 | 0.88 |
|  |  | 2344 | 297 | 304 | 96 | 1647 | 0.76 | 0.84 | 0.88 |
|  |  | 2495 | 103 | 94 | 98 | 2200 | 0.51 | 0.96 | 0.82 |
|  |  | 5370 | 104 | 554 | 92 | 4620 | 0.53 | 0.89 | 0.82 |
|  |  | 2112 | 97 | 291 | 64 | 1660 | 0.60 | 0.85 | 0.80 |
| Dhar et al. [8] | 2023 | 237 | 97 | 12 | 19 | 109 | 0.84 | 0.90 | 0.95 |
|  |  | 114 | 25 | 11 | 4 | 74 | 0.87 | 0.87 | 0.87 |
| Reilly et al. [35] | 2023 | 1453 | 18 | 180 | 4 | 1251 | 0.82 | 0.87 | 0.91 |
|  |  | 263 | 3 | 30 | 2 | 228 | 0.60 | 0.88 | NR |
|  |  | 138 | 0 | 15 | 2 | 121 | 0.00 | 0.89 | NR |
|  |  | 100 | 1 | 19 | 2 | 78 | 0.33 | 0.80 | NR |
|  |  | 83 | 32 | 2 | 6 | 43 | 0.84 | 0.96 | NR |
|  |  | 501 | 4 | 64 | 6 | 427 | 0.40 | 0.87 | NR |
| Chen et al. [31] | 2023 | 44 | 24 | 2 | 3 | 15 | 0.89 | 0.88 | 0.91 |
| Zhang et al. [36] | 2023 | 301 | 46 | 31 | 29 | 195 | 0.62 | 0.86 | 0.86 |
|  |  | 301 | 64 | 43 | 11 | 183 | 0.85 | 0.81 | 0.92 |
|  |  | 301 | 62 | 7 | 13 | 219 | 0.82 | 0.97 | 0.97 |
|  |  | 301 | 69 | 12 | 6 | 214 | 0.92 | 0.95 | 0.99 |
|  |  | 301 | 67 | 4 | 8 | 222 | 0.89 | 0.98 | 0.99 |
| Lai et al. [33] | 2023 | 545 | 178 | 30 | 118 | 219 | 0.60 | 0.88 | 0.81 |
|  |  | 233 | 65 | 30 | 62 | 77 | 0.51 | 0.72 | 0.61 |
|  |  | 778 | 243 | 62 | 180 | 293 | 0.57 | 0.83 | 0.75 |
| Bifarin et al. [29] | 2023 | 227 | 117 | 17 | 27 | 66 | 0.81 | 0.79 | 0.86 |
|  |  | 227 | 112 | 21 | 32 | 62 | 0.78 | 0.75 | 0.84 |
|  |  | 227 | 82 | 15 | 62 | 68 | 0.57 | 0.82 | 0.77 |
|  |  | 227 | 107 | 15 | 37 | 68 | 0.74 | 0.82 | 0.87 |
|  |  | 98 | 48 | 9 | 16 | 25 | 0.75 | 0.74 | 0.85 |
|  |  | 98 | 46 | 8 | 18 | 26 | 0.72 | 0.76 | 0.82 |
|  |  | 98 | 36 | 8 | 28 | 26 | 0.56 | 0.76 | 0.69 |
|  |  | 98 | 48 | 6 | 16 | 28 | 0.75 | 0.82 | 0.85 |
| Hamidi et al. [32] | 2023 | 2156 | 1035 | 0 | 0 | 1121 | 1.00 | 1.00 | 1.00 |
|  |  | 2156 | 1008 | 33 | 27 | 1088 | 0.97 | 0.97 | 0.98 |
|  |  | 2156 | 1030 | 0 | 5 | 1121 | 1.00 | 1.00 | 1.00 |
|  |  | 2156 | 1023 | 14 | 12 | 1107 | 0.99 | 0.99 | 1.00 |
|  |  | 2156 | 1028 | 0 | 7 | 1121 | 0.99 | 1.00 | 1.00 |
|  |  | 3079 | 317 | 60 | 3 | 2699 | 0.99 | 0.98 | 1.00 |
|  |  | 3079 | 297 | 40 | 23 | 2719 | 0.93 | 0.99 | 0.98 |
|  |  | 3079 | 307 | 14 | 13 | 2745 | 0.96 | 1.00 | 1.00 |
|  |  | 3079 | 313 | 30 | 7 | 2729 | 0.98 | 0.99 | 1.00 |
|  |  | 3079 | 307 | 17 | 13 | 2742 | 0.96 | 0.99 | 1.00 |
|  |  | 92 | 40 | 0 | 0 | 52 | 1.00 | 1.00 | 1.00 |
|  |  | 92 | 37 | 5 | 3 | 47 | 0.93 | 0.90 | 0.93 |
|  |  | 92 | 37 | 0 | 3 | 52 | 0.93 | 1.00 | 1.00 |
|  |  | 92 | 40 | 0 | 0 | 52 | 1.00 | 1.00 | 1.00 |
|  |  | 92 | 39 | 0 | 1 | 52 | 0.98 | 1.00 | 1.00 |
|  |  | 240 | 25 | 0 | 0 | 215 | 1.00 | 1.00 | 1.00 |
|  |  | 240 | 23 | 21 | 2 | 194 | 0.93 | 0.90 | 0.93 |
|  |  | 240 | 23 | 0 | 2 | 215 | 0.93 | 1.00 | 1.00 |
|  |  | 240 | 25 | 0 | 0 | 215 | 1.00 | 1.00 | 1.00 |
|  |  | 240 | 24 | 0 | 1 | 215 | 0.98 | 1.00 | 1.00 |
| Li et al. [34] | 2023 | 69 | 50 | 0 | 4 | 15 | 0.93 | 1.00 | 0.97 |
|  |  | 24 | 7 | 0 | 2 | 15 | 0.78 | 1.00 | 0.93 |
|  |  | 69 | 33 | 0 | 21 | 15 | 0.61 | 1.00 | 0.81 |
|  |  | 69 | 31 | 1 | 23 | 14 | 0.57 | 0.93 | 0.79 |
|  |  | 69 | 40 | 1 | 14 | 14 | 0.74 | 0.93 | 0.89 |
|  |  | 69 | 35 | 0 | 19 | 15 | 0.65 | 1.00 | 0.82 |
|  |  | 69 | 29 | 0 | 25 | 15 | 0.54 | 1.00 | 0.74 |
|  |  | 69 | 24 | 0 | 30 | 15 | 0.44 | 1.00 | 0.71 |
|  |  | 69 | 31 | 0 | 23 | 15 | 0.57 | 1.00 | 0.80 |
|  |  | 69 | 41 | 3 | 14 | 12 | 0.75 | 0.80 | 0.86 |
|  |  | 69 | 36 | 2 | 18 | 13 | 0.67 | 0.87 | 0.84 |
|  |  | 24 | 5 | 3 | 4 | 12 | 0.56 | 0.80 | 0.72 |
|  |  | 24 | 4 | 2 | 5 | 13 | 0.44 | 0.87 | 0.74 |
| Abuzinadah et al. [28] | 2023 | 349 | 146 | 26 | 25 | 152 | 0.85 | 0.86 | NR |
|  |  | 349 | 150 | 29 | 21 | 149 | 0.88 | 0.84 | NR |
|  |  | 349 | 147 | 28 | 24 | 150 | 0.86 | 0.84 | NR |
|  |  | 349 | 148 | 23 | 23 | 155 | 0.87 | 0.87 | NR |
|  |  | 349 | 150 | 24 | 21 | 154 | 0.88 | 0.87 | NR |
|  |  | 349 | 134 | 42 | 37 | 136 | 0.78 | 0.77 | NR |
|  |  | 349 | 146 | 24 | 25 | 154 | 0.86 | 0.86 | NR |
|  |  | 349 | 144 | 26 | 27 | 152 | 0.84 | 0.85 | NR |
|  |  | 349 | 151 | 21 | 20 | 157 | 0.88 | 0.88 | NR |
|  |  | 349 | 169 | 9 | 2 | 169 | 0.99 | 0.95 | NR |
|  |  | 105 | 50 | 0 | 1 | 53 | 0.98 | 1.00 | NR |
|  |  | 244 | 120 | 2 | 0 | 123 | 1.00 | 0.99 | NR |
| Cameron et al. [30] | 2023 | 385 | 115 | 120 | 10 | 140 | 0.92 | 0.54 | NR |
|  |  | 385 | 73 | 26 | 53 | 234 | 0.58 | 0.90 | NR |
| Ahamad et al. [36] | 2022 | 349 | 157 | 53 | 14 | 125 | 0.92 | 0.70 | 0.78 |
|  |  | 349 | 152 | 48 | 19 | 131 | 0.89 | 0.73 | 0.78 |
|  |  | 349 | 133 | 29 | 38 | 149 | 0.78 | 0.84 | 0.81 |
|  |  | 349 | 147 | 42 | 24 | 136 | 0.86 | 0.76 | 0.77 |
|  |  | 349 | 139 | 37 | 32 | 141 | 0.81 | 0.79 | 0.78 |
|  |  | 349 | 144 | 35 | 27 | 143 | 0.84 | 0.80 | 0.82 |
|  |  | 349 | 147 | 39 | 24 | 139 | 0.86 | 0.78 | 0.82 |
|  |  | 349 | 142 | 37 | 29 | 141 | 0.83 | 0.79 | 0.80 |
|  |  | 349 | 154 | 53 | 17 | 125 | 0.90 | 0.70 | 0.79 |
|  |  | 349 | 116 | 57 | 55 | 121 | 0.68 | 0.68 | 0.68 |
|  |  | 349 | 133 | 46 | 38 | 132 | 0.78 | 0.74 | 0.77 |
|  |  | 349 | 152 | 51 | 19 | 127 | 0.89 | 0.71 | 0.79 |
|  |  | 349 | 130 | 46 | 41 | 132 | 0.76 | 0.74 | 0.75 |
|  |  | 349 | 140 | 56 | 31 | 122 | 0.82 | 0.68 | 0.76 |
|  |  | 349 | 166 | 44 | 5 | 134 | 0.97 | 0.75 | 0.86 |
|  |  | 349 | 162 | 44 | 9 | 134 | 0.95 | 0.75 | 0.84 |
|  |  | 349 | 157 | 39 | 14 | 139 | 0.92 | 0.78 | 0.85 |
|  |  | 349 | 166 | 44 | 5 | 134 | 0.97 | 0.75 | 0.86 |
|  |  | 349 | 157 | 46 | 14 | 132 | 0.92 | 0.74 | 0.83 |
|  |  | 349 | 162 | 44 | 9 | 134 | 0.95 | 0.75 | 0.84 |
|  |  | 349 | 162 | 44 | 9 | 134 | 0.95 | 0.75 | 0.84 |
|  |  | 349 | 162 | 33 | 9 | 145 | 0.95 | 0.81 | 0.87 |
|  |  | 349 | 152 | 48 | 19 | 131 | 0.89 | 0.73 | 0.80 |
|  |  | 349 | 133 | 39 | 38 | 139 | 0.78 | 0.78 | 0.78 |
|  |  | 349 | 162 | 40 | 9 | 138 | 0.95 | 0.77 | 0.86 |
|  |  | 349 | 152 | 44 | 19 | 134 | 0.89 | 0.75 | 0.82 |
|  |  | 349 | 162 | 33 | 9 | 145 | 0.95 | 0.81 | 0.87 |
|  |  | 349 | 157 | 28 | 14 | 150 | 0.92 | 0.84 | 0.87 |
|  |  | 106 | 62 | 15 | 0 | 29 | 1.00 | 0.66 | 0.81 |
|  |  | 106 | 62 | 20 | 0 | 24 | 1.00 | 0.54 | 0.75 |
|  |  | 106 | 53 | 16 | 9 | 28 | 0.86 | 0.64 | 0.74 |
|  |  | 106 | 53 | 16 | 9 | 28 | 0.86 | 0.64 | 0.74 |
|  |  | 106 | 62 | 19 | 0 | 25 | 1.00 | 0.57 | 0.75 |
|  |  | 106 | 45 | 12 | 17 | 32 | 0.73 | 0.73 | 0.68 |
|  |  | 106 | 62 | 38 | 0 | 6 | 1.00 | 0.13 | 0.50 |
|  |  | 106 | 58 | 20 | 4 | 24 | 0.93 | 0.54 | 0.71 |
|  |  | 106 | 58 | 20 | 4 | 24 | 0.93 | 0.54 | 0.71 |
|  |  | 106 | 44 | 25 | 18 | 19 | 0.71 | 0.42 | 0.54 |
|  |  | 106 | 53 | 20 | 9 | 24 | 0.86 | 0.55 | 0.68 |
|  |  | 106 | 58 | 20 | 4 | 24 | 0.93 | 0.54 | 0.71 |
|  |  | 106 | 45 | 12 | 17 | 32 | 0.73 | 0.73 | 0.68 |
|  |  | 106 | 62 | 38 | 0 | 6 | 1.00 | 0.13 | 0.50 |
|  |  | 106 | 53 | 1 | 9 | 43 | 0.86 | 0.98 | 0.93 |
|  |  | 106 | 49 | 6 | 13 | 38 | 0.79 | 0.86 | 0.83 |
|  |  | 106 | 22 | 4 | 40 | 40 | 0.36 | 0.91 | 0.68 |
|  |  | 106 | 31 | 3 | 31 | 41 | 0.50 | 0.93 | 0.75 |
|  |  | 106 | 49 | 6 | 13 | 38 | 0.79 | 0.86 | 0.83 |
|  |  | 106 | 51 | 9 | 11 | 35 | 0.82 | 0.80 | 0.83 |
|  |  | 106 | 62 | 38 | 0 | 6 | 1.00 | 0.13 | 0.50 |
|  |  | 106 | 58 | 11 | 4 | 34 | 0.93 | 0.76 | 0.84 |
|  |  | 106 | 35 | 12 | 27 | 33 | 0.57 | 0.74 | 0.66 |
|  |  | 106 | 31 | 3 | 31 | 41 | 0.50 | 0.93 | 0.75 |
|  |  | 106 | 49 | 2 | 13 | 42 | 0.79 | 0.96 | 0.89 |
|  |  | 106 | 62 | 15 | 0 | 29 | 1.00 | 0.66 | 0.81 |
|  |  | 106 | 53 | 6 | 9 | 38 | 0.86 | 0.86 | 0.87 |
|  |  | 106 | 62 | 38 | 0 | 6 | 1.00 | 0.13 | 0.50 |
| Bahado-Singh et al. [38] | 2022 | 17 | 5 | 0 | 0 | 12 | 0.94 | 0.99 | 0.99 |
|  |  | 17 | 5 | 0 | 0 | 12 | 0.95 | 1.00 | 0.99 |
|  |  | 17 | 5 | 3 | 0 | 9 | 1.00 | 0.72 | 1.00 |
|  |  | 17 | 5 | 2 | 0 | 10 | 1.00 | 0.81 | 1.00 |
|  |  | 17 | 5 | 2 | 0 | 10 | 1.00 | 0.80 | 1.00 |
|  |  | 17 | 5 | 3 | 0 | 9 | 1.00 | 0.77 | 1.00 |
|  |  | 17 | 5 | 3 | 0 | 9 | 1.00 | 0.75 | 1.00 |
|  |  | 17 | 5 | 1 | 0 | 11 | 1.00 | 0.88 | 1.00 |
|  |  | 17 | 5 | 4 | 0 | 8 | 1.00 | 0.70 | 1.00 |
|  |  | 17 | 5 | 2 | 0 | 10 | 1.00 | 0.80 | 1.00 |
|  |  | 17 | 5 | 2 | 0 | 10 | 1.00 | 0.81 | 1.00 |
|  |  | 17 | 5 | 3 | 0 | 9 | 1.00 | 0.75 | 1.00 |
|  |  | 17 | 5 | 3 | 0 | 9 | 1.00 | 0.75 | 1.00 |
|  |  | 17 | 5 | 1 | 0 | 11 | 1.00 | 0.88 | 1.00 |
| Kim et al. [41] | 2022 | 215 | 53 | 8 | 3 | 151 | 0.94 | 0.95 | NR |
|  |  | 215 | 49 | 3 | 7 | 156 | 0.87 | 0.98 | NR |
| Gupta et al. [39] | 2022 | 681 | 113 | 39 | 18 | 512 | 0.86 | 0.93 | 0.92 |
| Hinestrosa et al. [40] | 2022 | 228 | 33 | 1 | 11 | 183 | 0.75 | 1.00 | NR |
| Irajizad et al. [25] | 2022 | 235 | 69 | 28 | 32 | 106 | 0.68 | 0.79 | 0.81 |
|  |  | 235 | 69 | 16 | 32 | 118 | 0.68 | 0.88 | 0.84 |
|  |  | 174 | 106 | 13 | 12 | 43 | 0.90 | 0.77 | 0.91 |
|  |  | 174 | 106 | 5 | 12 | 51 | 0.90 | 0.91 | 0.93 |
|  |  | 235 | 88 | 28 | 13 | 106 | 0.87 | 0.79 | 0.91 |
|  |  | 235 | 87 | 16 | 14 | 118 | 0.86 | 0.88 | 0.93 |
|  |  | 174 | 113 | 13 | 5 | 43 | 0.96 | 0.76 | 0.96 |
|  |  | 174 | 110 | 5 | 8 | 51 | 0.93 | 0.91 | 0.97 |
|  |  | 409 | 166 | 42 | 53 | 148 | 0.76 | 0.78 | 0.84 |
|  |  | 409 | 166 | 21 | 53 | 169 | 0.76 | 0.89 | 0.87 |
| Li et al. [42] | 2022 | 178 | 64 | 4 | 14 | 96 | 0.82 | 0.96 | NR |
|  |  | 184 | 70 | 5 | 15 | 94 | 0.82 | 0.95 | NR |
| Pais et al. [43] | 2022 | 181 | 134 | 11 | 9 | 27 | 0.94 | 0.71 | NR |
|  |  | 181 | 141 | 3 | 2 | 35 | 0.99 | 0.92 | NR |
| Jeong et al. [44] | 2021 | 730 | 40 | 211 | 13 | 466 | 0.75 | 0.69 | 0.76 |
| Lu et al. [45] | 2020 | 235 | 108 | 10 | 38 | 79 | 0.74 | 0.89 | 0.81 |
|  |  | 235 | 120 | 4 | 26 | 85 | 0.82 | 0.96 | 0.89 |
|  |  | 235 | 110 | 0 | 36 | 89 | 0.75 | 1.00 | 0.88 |
|  |  | 114 | 25 | 9 | 0 | 80 | 1.00 | 0.90 | 0.94 |
|  |  | 114 | 23 | 3 | 2 | 86 | 0.92 | 0.97 | 0.95 |
|  |  | 114 | 24 | 2 | 1 | 87 | 0.96 | 0.98 | 0.97 |
| Banaei et al. [46] | 2019 | 10 | 4 | 1 | 1 | 4 | 0.80 | 0.80 | NR |
|  |  | 10 | 2 | 0 | 3 | 5 | 0.40 | 0.93 | NR |
|  |  | 10 | 4 | 0 | 1 | 5 | 0.80 | 1.00 | NR |
|  |  | 10 | 4 | 0 | 1 | 5 | 0.80 | 0.93 | NR |
|  |  | 10 | 4 | 0 | 1 | 5 | 0.80 | 0.93 | NR |
| Whitwell et al. [47] | 2018 | 59 | 5 | 0 | 24 | 30 | 0.19 | 1.00 | 0.62 |
|  |  | 59 | 5 | 0 | 24 | 30 | 0.16 | 1.00 | 0.66 |
|  |  | 60 | 24 | 0 | 6 | 30 | 0.80 | 1.00 | 0.90 |
|  |  | 60 | 23 | 0 | 7 | 30 | 0.77 | 1.00 | 0.90 |
| Ivanova et al. [48] | 2016 | 67 | 34 | 0 | 0 | 33 | 1.00 | 1.00 | NR |
| Jiang et al. [49] | 2013 | 87 | 34 | 3 | 0 | 50 | 1.00 | 0.95 | 0.98 |
| Yang et al. [50] | 2013 | 23 | 12 | 1 | 0 | 10 | 1.00 | 0.91 | NR |
|  |  | 31 | 10 | 1 | 1 | 19 | 0.91 | 0.95 | NR |
|  |  | 25 | 13 | 2 | 0 | 10 | 1.00 | 0.83 | NR |
| Shan et al. [51] | 2012 | 423 | 170 | 64 | 41 | 148 | 0.81 | 0.70 | NR |
|  |  | 423 | 170 | 64 | 41 | 148 | 0.81 | 0.70 | NR |
|  |  | 423 | 194 | 96 | 17 | 116 | 0.92 | 0.55 | NR |
|  |  | 423 | 160 | 60 | 51 | 152 | 0.76 | 0.72 | NR |
|  |  | 423 | 160 | 60 | 51 | 152 | 0.76 | 0.72 | NR |
| Thakur et al. [52] | 2011 | 216 | 103 | 28 | 18 | 67 | 0.85 | 0.71 | NR |
|  |  | 216 | 119 | 4 | 2 | 91 | 0.98 | 0.96 | NR |
| Donach et al. [53] | 2010 | 187 | 50 | 10 | 7 | 120 | 0.88 | 0.93 | 0.89 |
| Ziganshin et al. [54] | 2008 | 94 | 41 | 2 | 6 | 45 | 0.88 | 0.96 | NR |
|  |  | 94 | 46 | 8 | 1 | 39 | 0.98 | 0.83 | NR |
|  |  | 94 | 46 | 6 | 1 | 41 | 0.97 | 0.88 | NR |
|  |  | 94 | 47 | 0 | 0 | 47 | 1.00 | 1.00 | NR |
|  |  | 94 | 41 | 1 | 6 | 46 | 0.88 | 0.98 | NR |
|  |  | 94 | 38 | 0 | 9 | 47 | 0.81 | 1.00 | NR |
|  |  | 94 | 47 | 5 | 0 | 42 | 1.00 | 0.90 | NR |
|  |  | 94 | 47 | 0 | 0 | 47 | 1.00 | 1.00 | NR |
| Liu et al. [55] | 2007 | 78 | 63 | 0 | 0 | 15 | 1.00 | 1.00 | NR |
|  |  | 78 | 63 | 0 | 0 | 15 | 1.00 | 1.00 | NR |
|  |  | 78 | 63 | 0 | 0 | 15 | 1.00 | 1.00 | NR |
|  |  | 78 | 63 | 1 | 0 | 14 | 1.00 | 0.93 | NR |
|  |  | 77 | 62 | 0 | 0 | 15 | 1.00 | 1.00 | NR |
|  |  | 77 | 62 | 0 | 0 | 15 | 1.00 | 1.00 | NR |
|  |  | 77 | 62 | 0 | 0 | 15 | 1.00 | 1.00 | NR |
|  |  | 77 | 62 | 0 | 0 | 15 | 1.00 | 1.00 | NR |
|  |  | 170 | 34 | 0 | 1 | 135 | 0.99 | 1.00 | NR |
|  |  | 170 | 32 | 0 | 4 | 131 | 0.98 | 1.00 | NR |
| Zhang et al. [56] | 2007 | 150 | 37 | 2 | 15 | 96 | 0.71 | 0.98 | 0.94 |
| Chatterjee et al. [57] | 2006 | 44 | 13 | 0 | 11 | 20 | 0.55 | 0.98 | NR |
|  |  | 85 | 14 | 2 | 31 | 38 | 0.32 | 0.94 | NR |
| Lin et al. [58] | 2006 | 65 | 27 | 0 | 3 | 35 | 0.90 | 1.00 | NR |
|  |  | 56 | 26 | 0 | 1 | 29 | 0.96 | 1.00 | NR |
| Liu et al. [59] | 2006 | 253 | 160 | 7 | 2 | 84 | 0.99 | 0.92 | NR |
|  |  | 253 | 162 | 19 | 0 | 72 | 1.00 | 0.79 | NR |
|  |  | 253 | 162 | 91 | 0 | 0 | 1.00 | 0.00 | NR |
| Wu et al. [60] | 2006 | 65 | 27 | 0 | 3 | 35 | 0.90 | 1.00 | NR |
|  |  | 65 | 25 | 4 | 5 | 31 | 0.84 | 0.89 | NR |
| Li et al. [61] | 2004 | 253 | 162 | 0 | 0 | 91 | 1.00 | 1.00 | NR |
|  |  | 253 | 158 | 6 | 4 | 85 | 0.98 | 0.93 | NR |
|  |  | 253 | 159 | 4 | 3 | 87 | 0.98 | 0.96 | NR |
|  |  | 253 | 158 | 6 | 4 | 85 | 0.98 | 0.93 | NR |
|  |  | 253 | 162 | 0 | 0 | 91 | 1.00 | 1.00 | NR |
|  |  | 253 | 157 | 10 | 5 | 81 | 0.97 | 0.89 | NR |
|  |  | 253 | 145 | 2 | 17 | 89 | 0.90 | 0.98 | NR |
| Li et al. [62] | 2004 | 216 | 79 | 23 | 21 | 93 | 0.79 | 0.80 | 0.80 |
|  |  | 216 | 98 | 6 | 2 | 110 | 0.98 | 0.95 | 0.96 |
|  |  | 253 | 160 | 3 | 2 | 88 | 0.99 | 0.97 | 0.98 |
|  |  | 216 | 96 | 6 | 4 | 110 | 0.96 | 0.95 | 0.95 |
|  |  | 216 | 98 | 1 | 2 | 115 | 0.98 | 0.99 | 0.99 |
|  |  | 253 | 162 | 0 | 0 | 91 | 1.00 | 1.00 | 1.00 |
|  |  | 216 | 79 | 23 | 21 | 93 | 0.79 | 0.80 | 0.80 |
|  |  | 216 | 96 | 6 | 4 | 110 | 0.96 | 0.95 | 0.95 |
|  |  | 216 | 47 | 19 | 53 | 97 | 0.47 | 0.84 | 0.67 |
| Zhang et al. [63] | 1999 | 167 | 72 | 7 | 9 | 79 | 0.89 | 0.92 | NR |
|  |  | 68 | 20 | 5 | 0 | 43 | 1.00 | 0.90 | NR |
| Wilding et al. [64] | 1994 | 98 | 21 | 16 | 14 | 47 | 0.61 | 0.74 | NR |
|  |  | 98 | 26 | 9 | 9 | 54 | 0.75 | 0.86 | NR |
|  |  | 98 | 28 | 11 | 7 | 52 | 0.81 | 0.82 | NR |
|  |  | 98 | 27 | 8 | 8 | 55 | 0.78 | 0.87 | NR |
|  |  | 98 | 28 | 9 | 7 | 54 | 0.81 | 0.86 | NR |
|  |  | 98 | 25 | 7 | 10 | 56 | 0.72 | 0.89 | NR |
|  |  | 98 | 28 | 11 | 7 | 52 | 0.81 | 0.82 | NR |
|  |  | 98 | 26 | 22 | 9 | 41 | 0.75 | 0.65 | NR |

Abbreviation: AUROC: area under the receiver operating characteristic curve; FP: false-positive; FN: false-negative; NR=not reported; TP: true-positive; TN: true-negative.
