# Supplementary material for: AI-Derived Blood Biomarkers for Ovarian Cancer Diagnosis: Systematic Review and Meta-Analysis
Source: J Med Internet Res. 2025 Mar 24;27:e67922. doi: 10.2196/67922 (PMC11976184; doi:10.2196/67922)
Supplement: Multimedia Appendix 4 [file jmir_v27i1e67922_app4.docx]

**[Multimedia Appendix](https://pmc.ncbi.nlm.nih.gov/articles/PMC10007007/#app1) 4. The list of the excluded records during the process of full-text review.**

**Incomplete data (n=5)**

77. Jing B, Chen G, Yang M, Zhang Z, Zhang Y, Zhang J, Xie J, Hou W, Xie Y, Huang Y, Zhao L, Yuan H, Liao W, Wang Y. Development of prediction model to estimate future risk of ovarian lesions: A multi-center retrospective study. Prev Med Rep 2023 Oct;35:102296. PMID:37455762

78. Lu H, Liu Y, Wang J, Fu S, Wang L, Huang C, Li J, Xie L, Wang D, Li D, Zhou H, Rao Q. Detection of ovarian cancer using plasma cell-free DNA methylomes. Clin Epigenetics 2022 Jun 9;14(1):74. PMID:35681212

79. Kawakami E, Tabata J, Yanaihara N, Ishikawa T, Koseki K, Iida Y, Saito M, Komazaki H, Shapiro JS, Goto C, Akiyama Y, Saito R, Saito M, Takano H, Yamada K, Okamoto A. Application of Artificial Intelligence for Preoperative Diagnostic and Prognostic Prediction in Epithelial Ovarian Cancer Based on Blood Biomarkers. Clin Cancer Res Off J Am Assoc Cancer Res 2019 May 15;25(10):3006–3015. PMID:30979733

80. Song H-J, Yang E-S, Kim J-D, Park C-Y, Kyung M-S, Kim Y-S. Best serum biomarker combination for ovarian cancer classification. Biomed Eng Online 2018 Nov 6;17(Suppl 2):152. PMID:30396341

81. Elias KM, Fendler W, Stawiski K, Fiascone SJ, Vitonis AF, Berkowitz RS, Frendl G, Konstantinopoulos P, Crum CP, Kedzierska M, Cramer DW, Chowdhury D. Diagnostic potential for a serum miRNA neural network for detection of ovarian cancer. eLife 2017 Oct 31;6:e28932. PMID:29087294

**Non-ovarian cancer (n=4)**

82. González G, Lakatos K, Hoballah J, Fritz-Klaus R, Al-Johani L, Brooker J, Jeong S, Evans CL, Krauledat P, Cramer DW, Hoffman RA, Hansen WP, Patankar MS. Characterization of Cell-Bound CA125 on Immune Cell Subtypes of Ovarian Cancer Patients Using a Novel Imaging Platform. Cancers 2021 Apr 25;13(9):2072. PMID:33922973

83. Gao Q, Lin YP, Li BS, Wang GQ, Dong LQ, Shen BY, Lou WH, Wu WC, Ge D, Zhu QL, Xu Y, Xu JM, Chang WJ, Lan P, Zhou PH, He MJ, Qiao GB, Chuai SK, Zang RY, Shi TY, Tan LJ, Yin J, Zeng Q, Su XF, Wang ZD, Zhao XQ, Nian WQ, Zhang S, Zhou J, Cai SL, Zhang ZH, Fan J. Unintrusive multi-cancer detection by circulating cell-free DNA methylation sequencing (THUNDER): development and independent validation studies. Ann Oncol Off J Eur Soc Med Oncol 2023 May;34(5):486–495. PMID:36849097

84. Ramamoorthy, S., Sundaramoorthy, S., Gupta, A. et al. Beta-Validation of a Non-Invasive Method for Simultaneous Detection of Early-Stage Female-Specific Cancers. medRxiv. Preprint posted online October 6, 2023. doi: 10.1101/2023.10.06.23296638

85. Luan Y, Zhong G, Li S, Wu W, Liu X, Zhu D, Feng Y, Zhang Y, Duan C, Mao M. A panel of seven protein tumour markers for effective and affordable multi-cancer early detection by artificial intelligence: a large-scale and multicentre case-control study. EClinicalMedicine 2023 Jul;61:102041. PMID:37387788

**Non-blood sample (n=3)**

86. Marcišauskas S, Ulfenborg B, Kristjansdottir B, Waldemarson S, Sundfeldt K. Univariate and classification analysis reveals potential diagnostic biomarkers for early stage ovarian cancer Type 1 and Type 2. J Proteomics 2019 Mar 30;196:57–68. PMID:30710757

87. Chiappa V, Interlenghi M, Bogani G, Salvatore C, Bertolina F, Sarpietro G, Signorelli M, Ronzulli D, Castiglioni I, Raspagliesi F. A decision support system based on radiomics and machine learning to predict the risk of malignancy of ovarian masses from transvaginal ultrasonography and serum CA-125. Eur Radiol Exp 2021 Jul 26;5(1):28. PMID:34308487

88. Hwangbo S, Kim SI, Kim J-H, Eoh KJ, Lee C, Kim YT, Suh D-S, Park T, Song YS. Development of Machine Learning Models to Predict Platinum Sensitivity of High-Grade Serous Ovarian Carcinoma. Cancers 2021 Apr 14;13(8):1875. PMID:33919797

**Non-diagnostic studies (n=3)**

1. Akazawa M, Hashimoto K. Artificial Intelligence in Ovarian Cancer Diagnosis. Anticancer Res 2020 Aug;40(8):4795–4800. PMID:32727807
2. Wu M, Zhao Y, Dong X, Jin Y, Cheng S, Zhang N, Xu S, Gu S, Wu Y, Yang J, Yao L, Wang Y. Artificial intelligence-based preoperative prediction system for diagnosis and prognosis in epithelial ovarian cancer: A multicenter study. Front Oncol 2022;12:975703. PMID:36212430
3. Yue Z, Sun C, Chen F, Zhang Y, Xu W, Shabbir S, Zou L, Lu W, Wang W, Xie Z, Zhou L, Lu Y, Yu J. Machine learning-based LIBS spectrum analysis of human blood plasma allows ovarian cancer diagnosis. Biomed Opt Express 2021 May 1;12(5):2559–2574. PMID:34123488
