# Supplementary material for: AI-Derived Blood Biomarkers for Ovarian Cancer Diagnosis: Systematic Review and Meta-Analysis
Source: J Med Internet Res. 2025 Mar 24;27:e67922. doi: 10.2196/67922 (PMC11976184; doi:10.2196/67922)
Supplement: Multimedia Appendix 5 [file jmir_v27i1e67922_app5.docx]

**
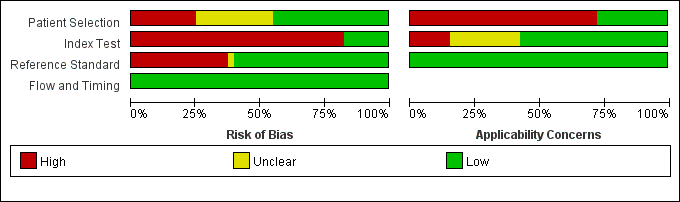
**

**Figure S1. Quality Assessment of Diagnostic Accuracy Studies-AI (QUADAS-AI) summary plot**


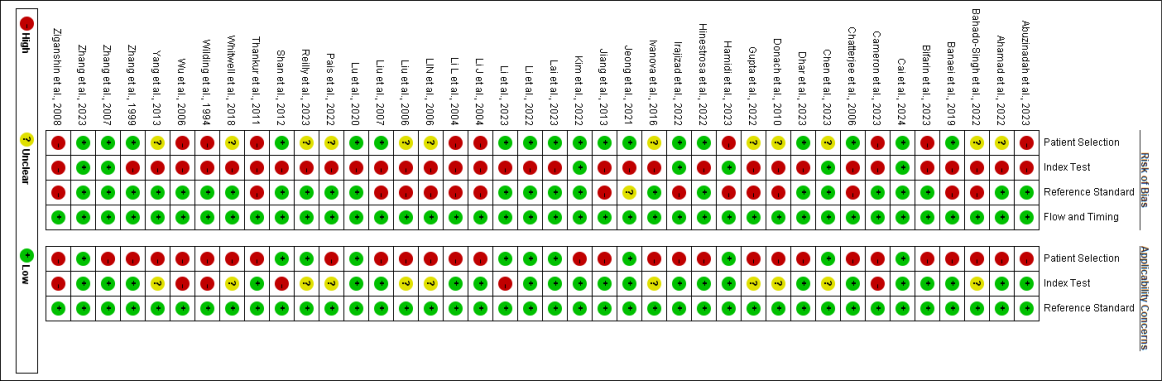


**Figure S2. Risk of bias and concern of applicability for each item in included studies**


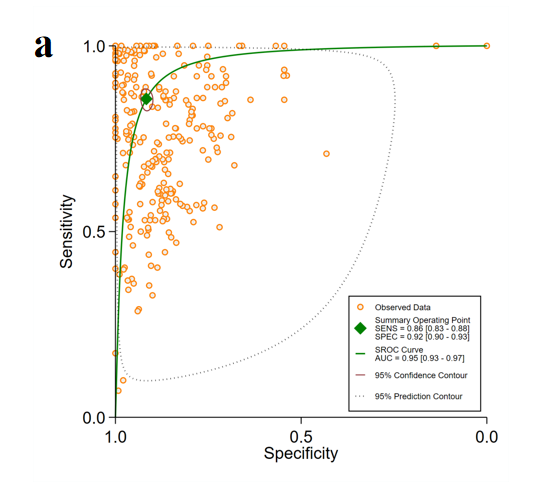

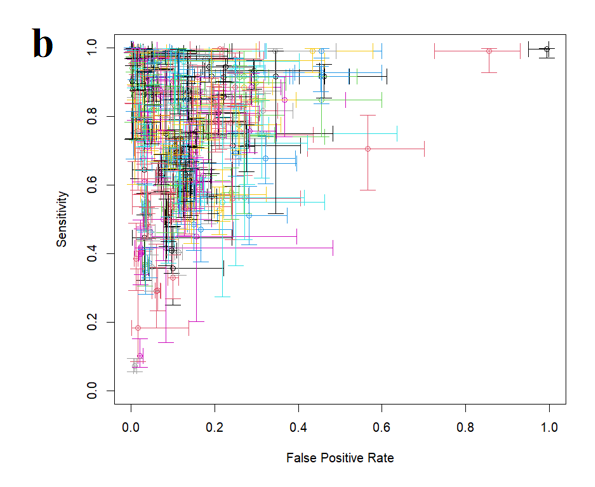

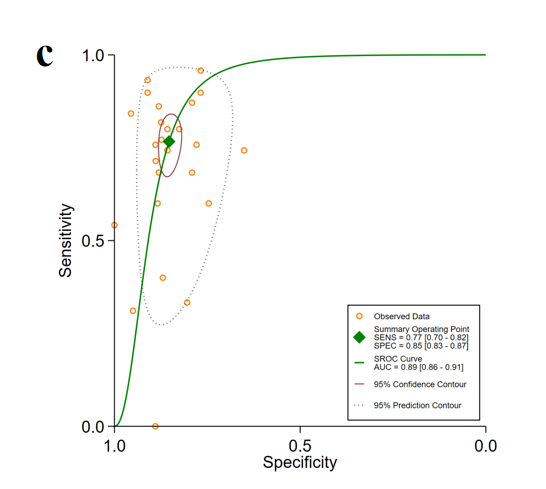

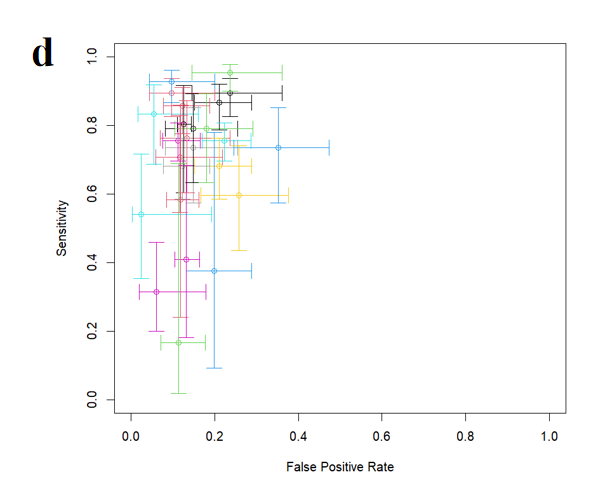


**Figure S3. SROC curve and Cross-hair plot of artificial intelligence algorithms (Machine learning, Deep learning)**

a: SROC curve of machine learning (36 studies with 316 tables)

b: Cross-hair plot of machine learning (36 studies with 316 tables)

c: SROC curve of deep learning (4 studies with 26 tables)

d: Cross-hair plot of deep learning (4 studies with 26 tables)

Abbreviations: SROC=summary receiver operating characteristic; SENS=summary sensitivity; SPEC=summary specificity.


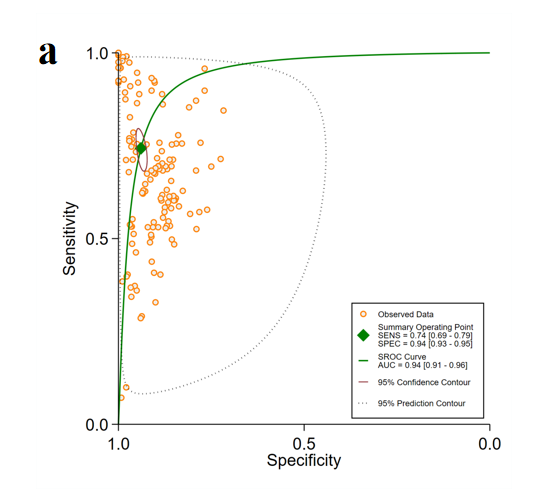

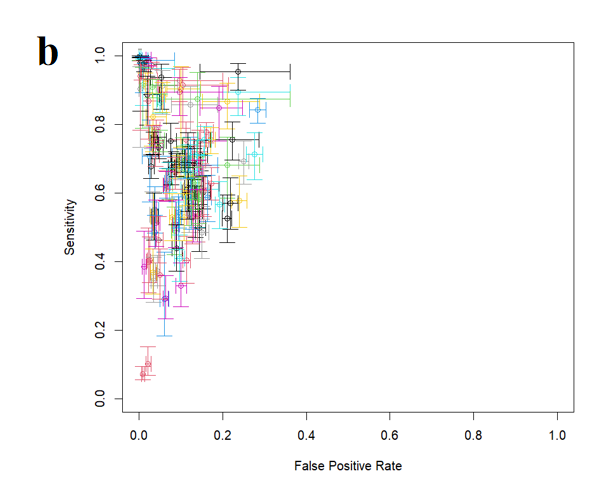

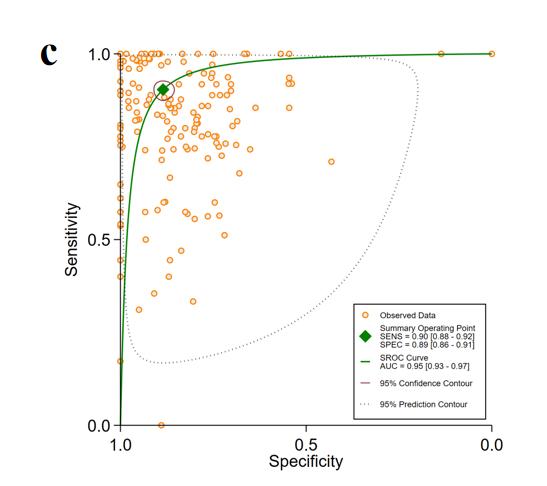

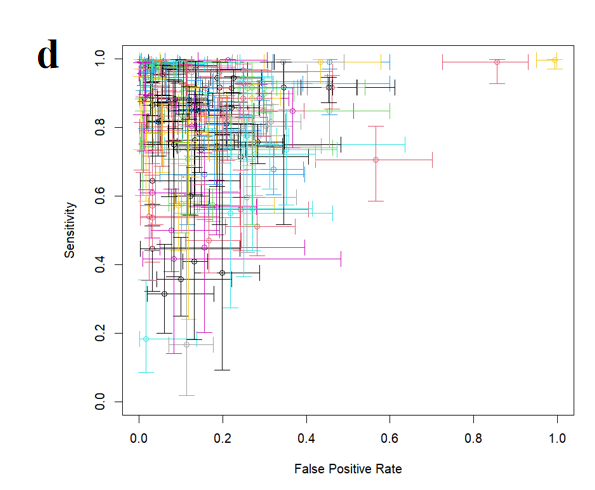


**Figure S4. SROC curve and Cross-hair plot of external validation (Yes, No)**

a: SROC curve of yes (7 studies with 138 tables)

b: Cross-hair plot of yes (7 studies with 138 tables)

c: SROC curve of no (33 studies with 204 tables)

d: Cross-hair plot of no (33 studies with 204 tables)

Abbreviations: SROC=summary receiver operating characteristic; SENS=summary sensitivity; SPEC=summary specificity.


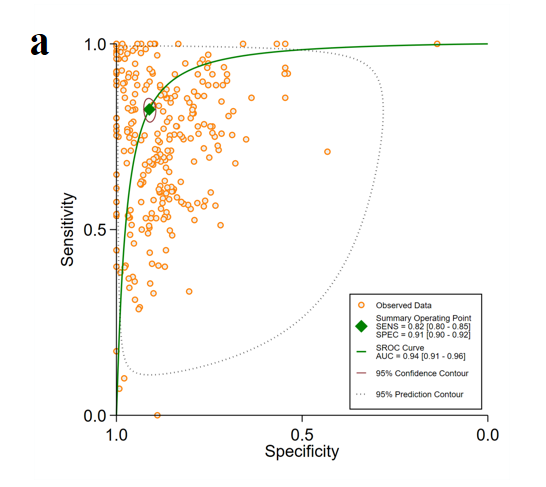

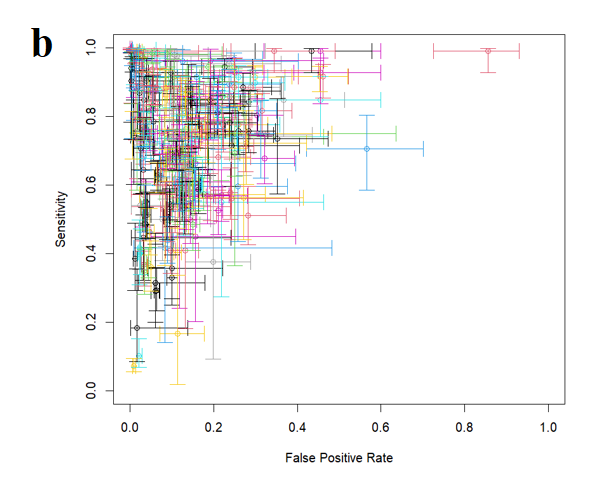

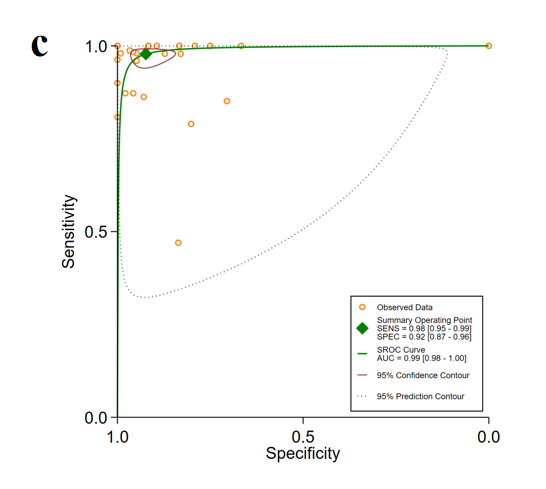

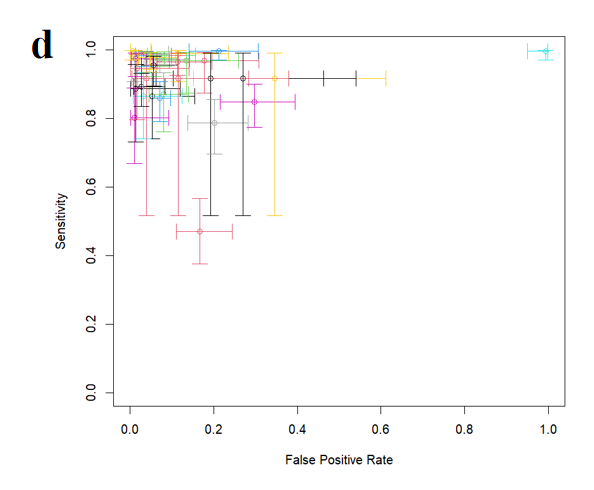


**Figure S5. SROC curve and Cross-hair plot of risk of bias levels (Low, High)**

a: SROC curve of low (31 studies with 295 tables)

b: Cross-hair plot of low (31 studies with 295 tables)

c: SROC curve of high (9 studies with 47 tables)

d: Cross-hair plot of high (9 studies with 47 tables)

Abbreviations: SROC=summary receiver operating characteristic; SENS=summary sensitivity; SPEC=summary specificity.


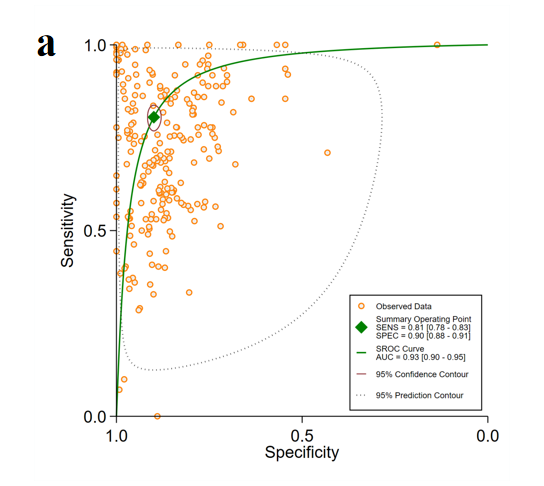

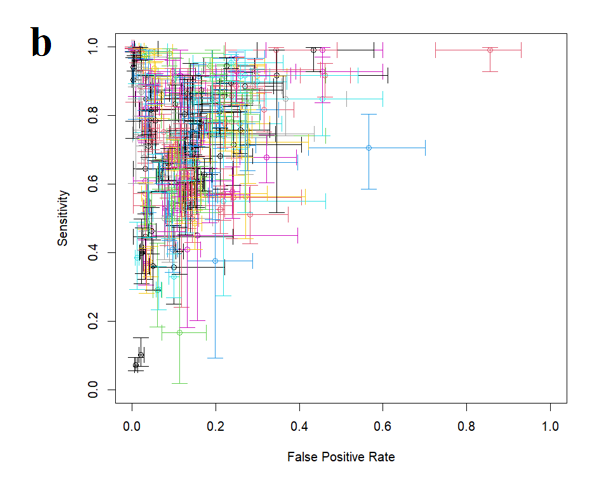

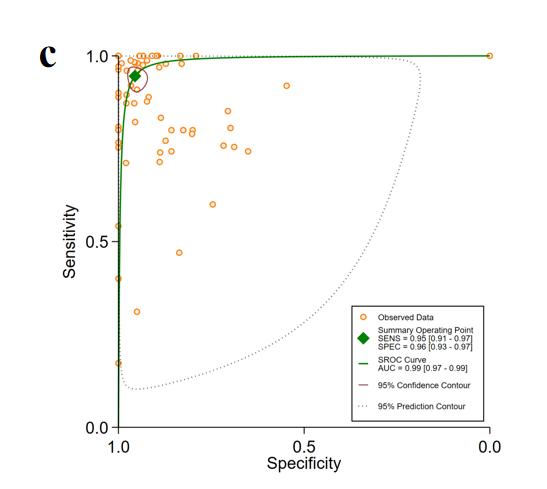

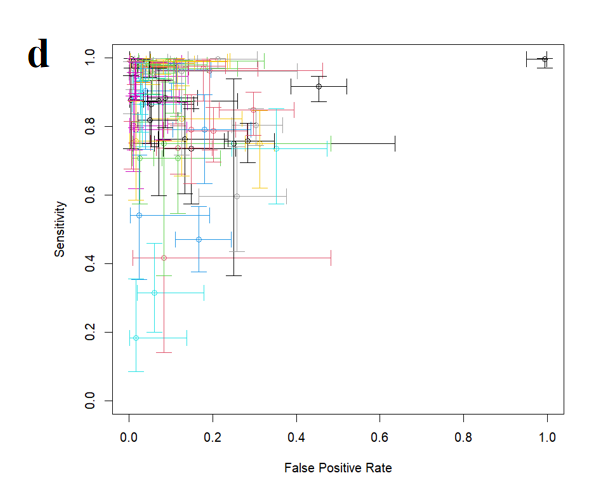


**Figure S6. SROC curve and Cross-hair plot of year of publication (After 2022, Before 2022)**

a: SROC curve of after 2022 (19 studies with 259 tables)

b: Cross-hair plot of after 2022 (19 studies with 259 tables)

c: SROC curve of before 2022 (21 studies with 83 tables)

d: Cross-hair plot of before 2022 (21 studies with 83 tables)

Abbreviations: SROC=summary receiver operating characteristic; SENS=summary sensitivity; SPEC=summary specificity.


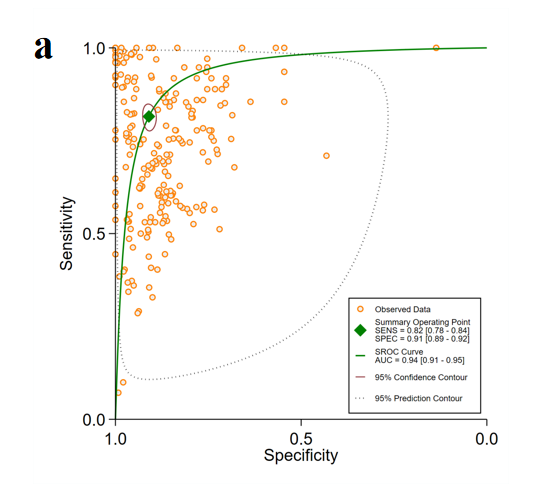

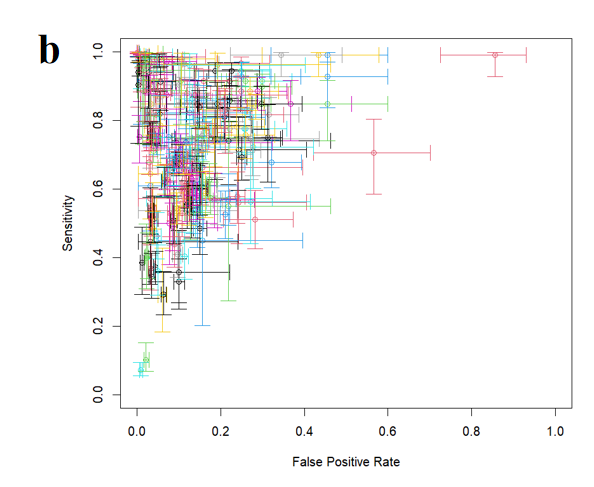

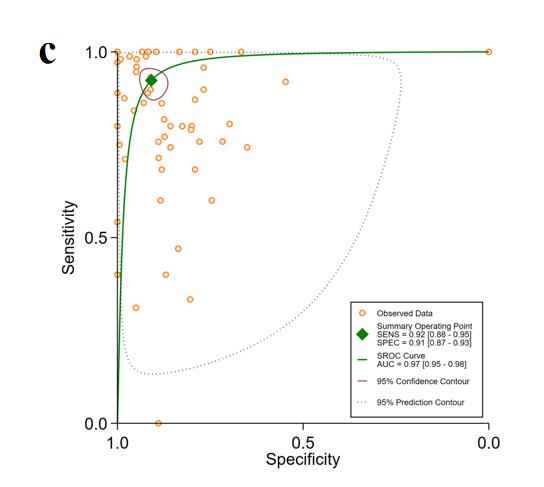

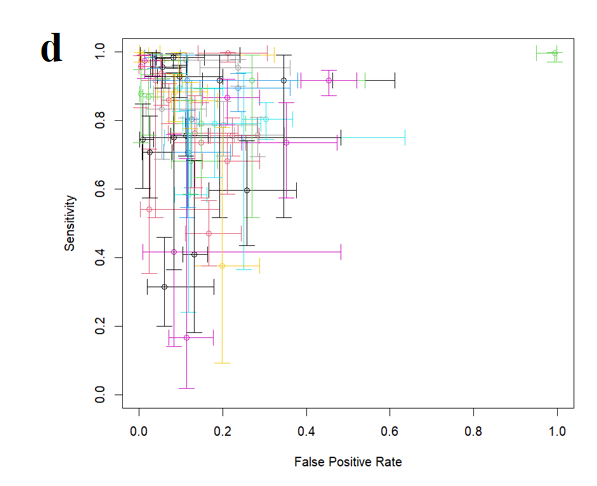

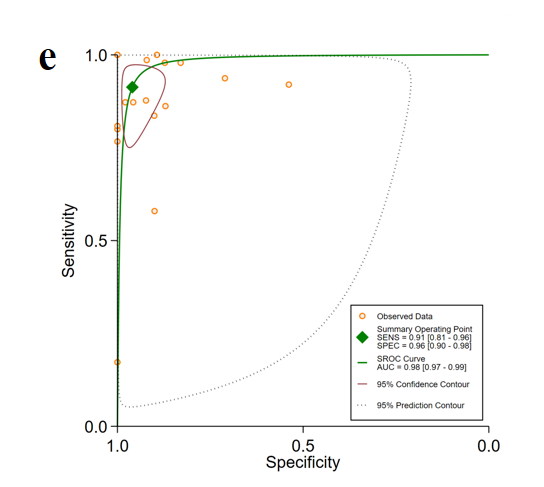

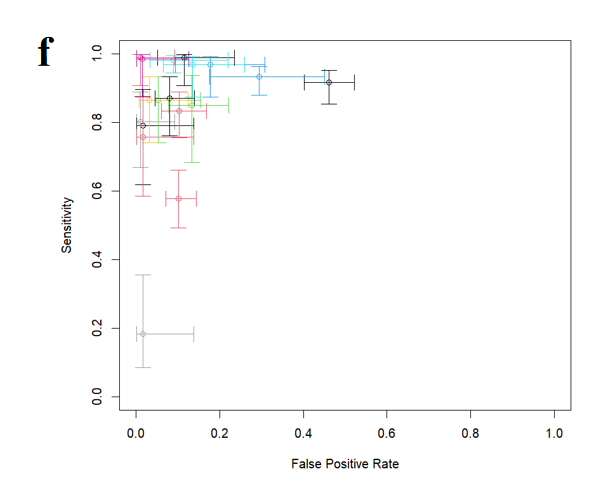


**Figure S7. SROC curve and Cross-hair plot of geographical distribution (Asia, North America, Europe)**

a: SROC curve of Asia (18 studies with 243 tables)

b: Cross-hair plot of Asia (18 studies with 243 tables)

c: SROC curve of North America (15 studies with 79 tables)

d: Cross-hair plot of North America (15 studies with 79 tables)

e: SROC curve of Europe (7 studies with 20 tables)

f: Cross-hair plot of Europe (7 studies with 20 tables)

Abbreviations: SROC=summary receiver operating characteristic; SENS=summary sensitivity; SPEC=summary specificity.


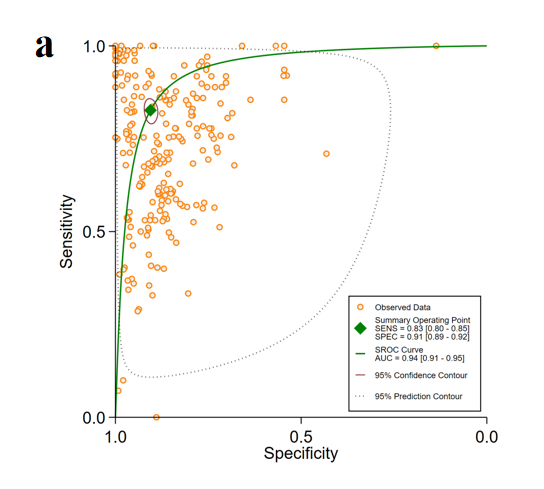

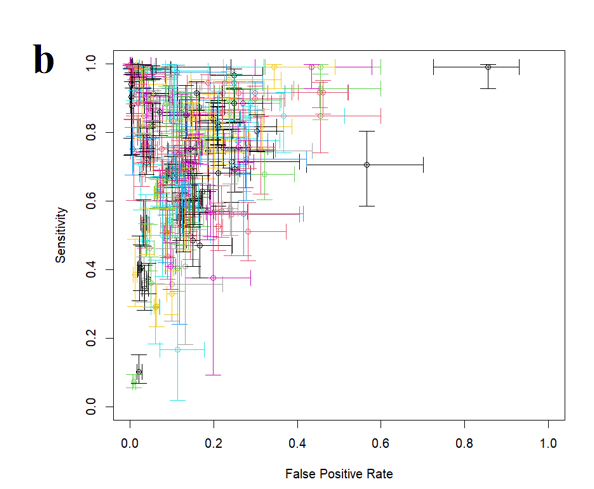

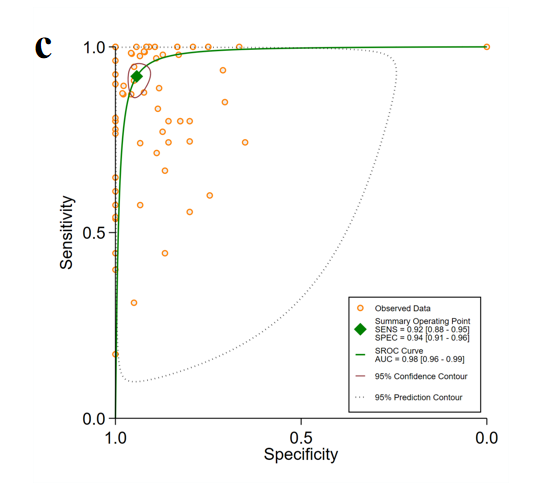

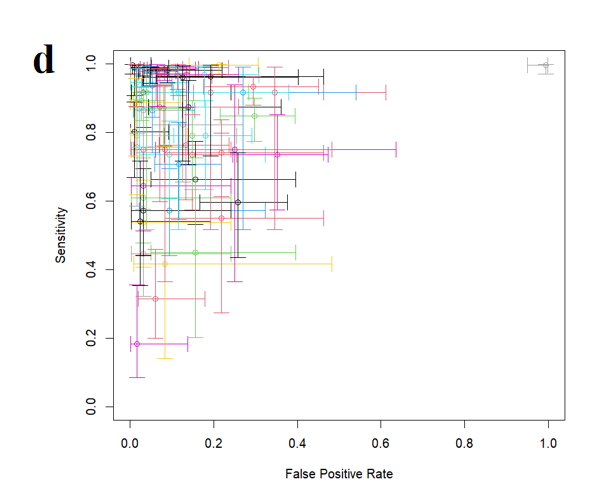


**Figure S8. SROC curve and Cross-hair plot of sample size (> 300, ≤ 300)**

a: SROC curve of sample size > 300 (21 studies with 261 tables)

b: Cross-hair plot of sample size > 300 (21 studies with 261 tables)

c: SROC curve of sample size ≤ 300 (19 studies with 81 tables)

d: Cross-hair plot of sample size ≤ 300 (19 studies with 81 tables)

Abbreviations: SROC=summary receiver operating characteristic; SENS=summary sensitivity; SPEC=summary specificity.


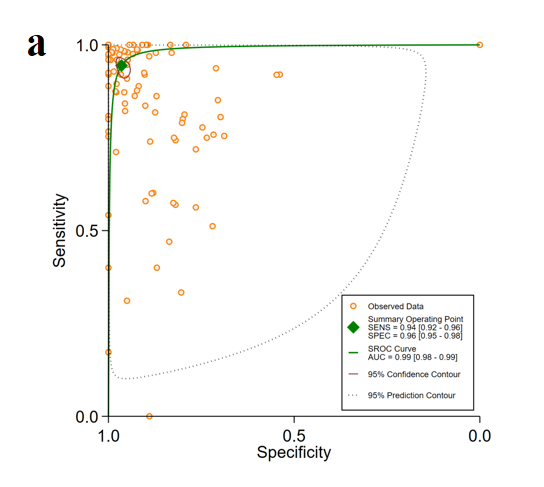

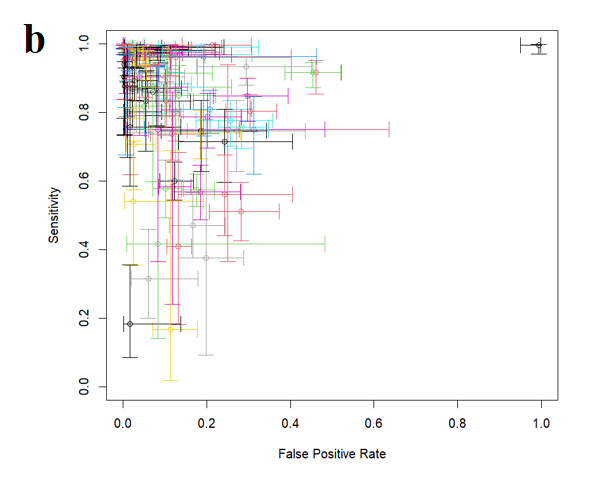

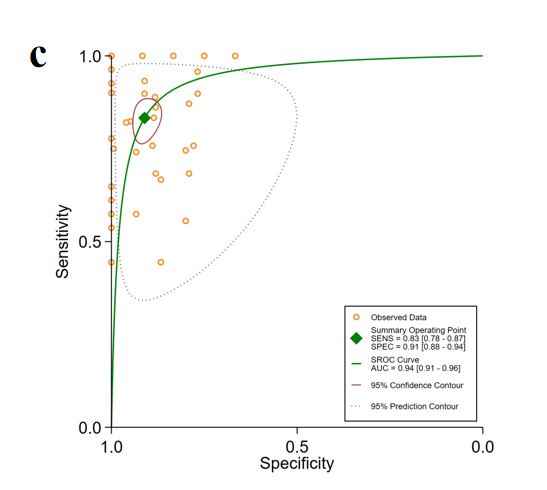

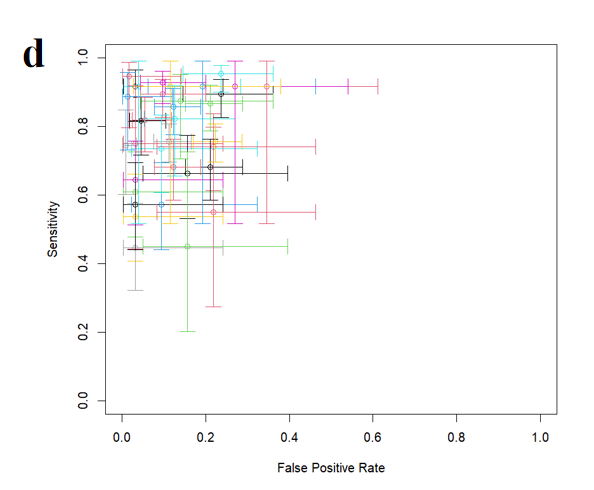


**Figure S9. SROC curve and Cross-hair plot of blood sample type (Serum, Plasma)**

a: SROC curve of serum (27 studies with 117 tables)

b: Cross-hair plot of serum (27 studies with 117 tables)

c: SROC curve of plasma (8 studies with 45 tables)

d: Cross-hair plot of plasma (8 studies with 45 tables)

Abbreviations: SROC=summary receiver operating characteristic; SENS=summary sensitivity; SPEC=summary specificity.


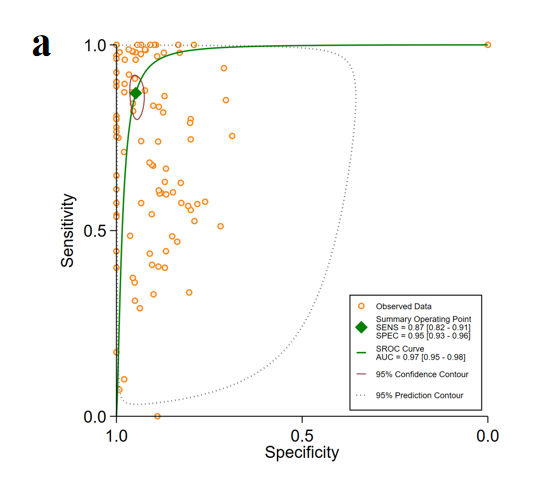

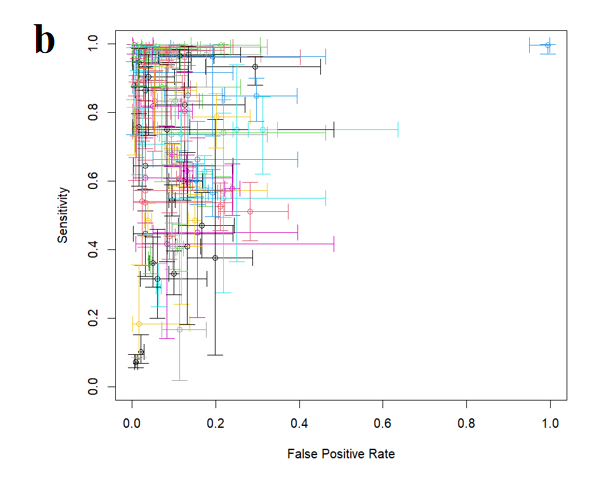

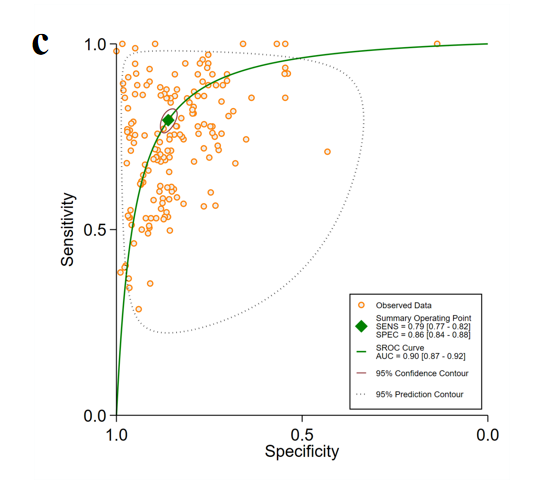

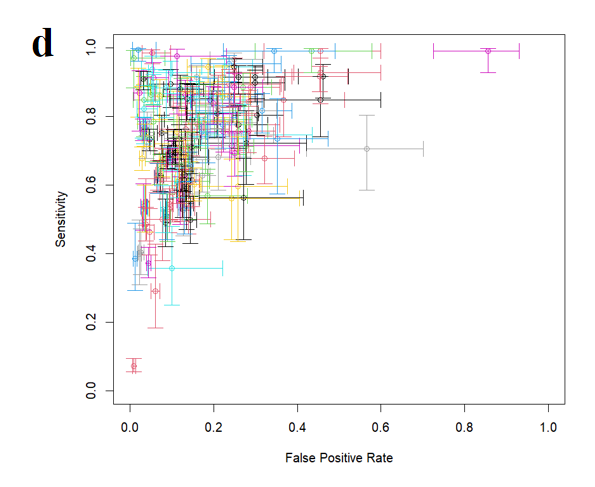


**Figure S10. SROC curve and Cross-hair plot of biomarker type (Protein, Mixed)**

a: SROC curve of protein (25 studies with 119 tables)

b: Cross-hair plot of protein (25 studies with 119 tables)

c: SROC curve of mixed (12 studies with 186 tables)

d: Cross-hair plot of mixed (12 studies with 186 tables)

Abbreviations: SROC=summary receiver operating characteristic; SENS=summary sensitivity; SPEC=summary specificity.


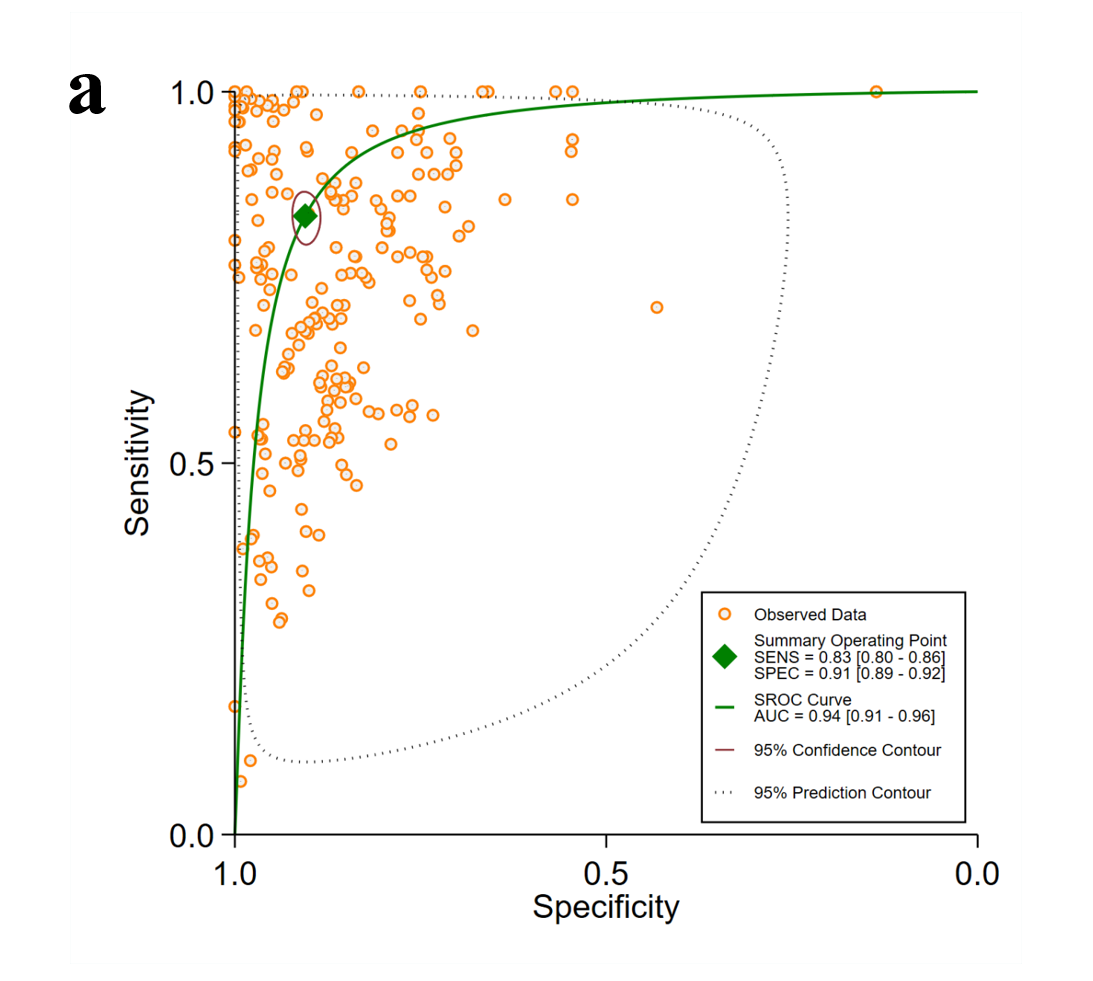

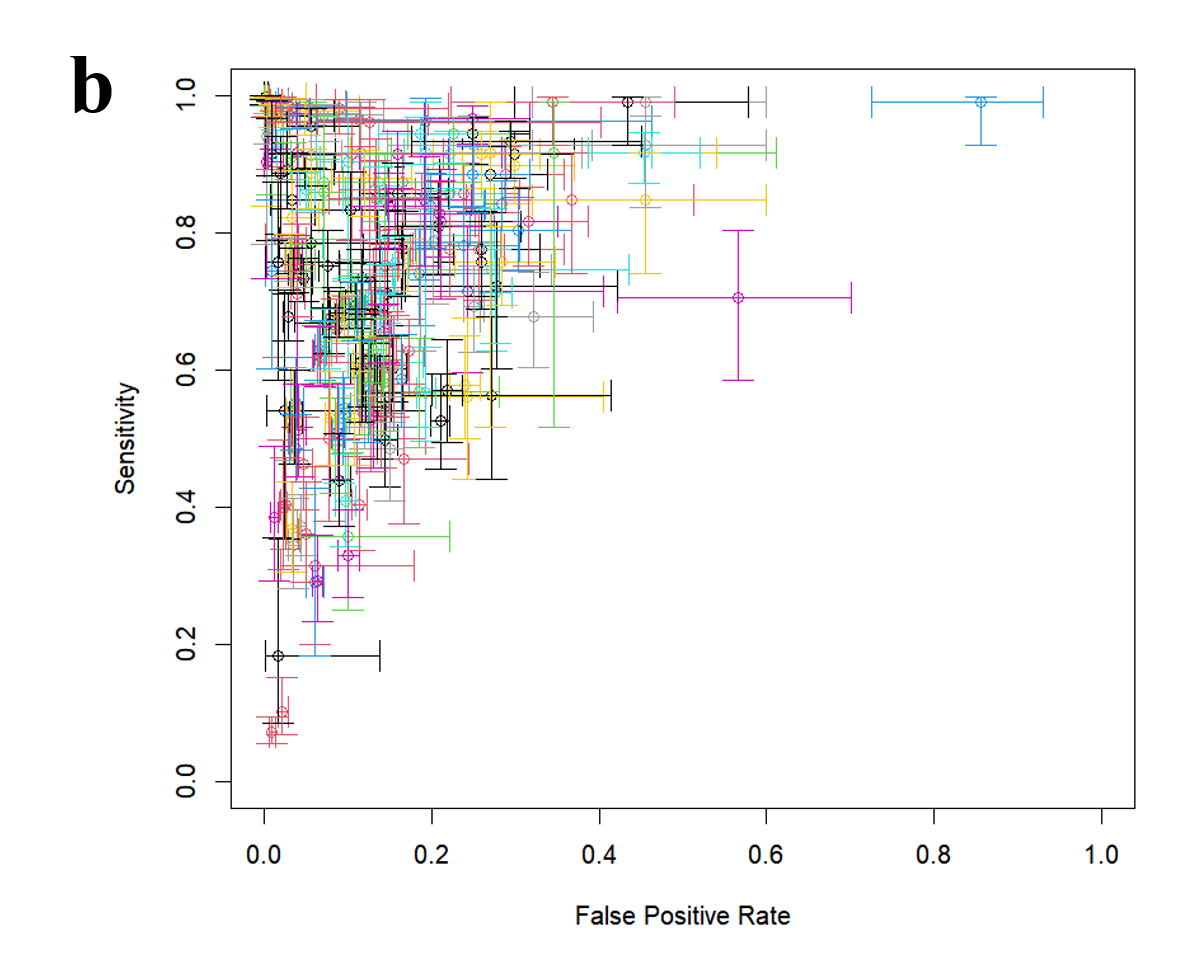

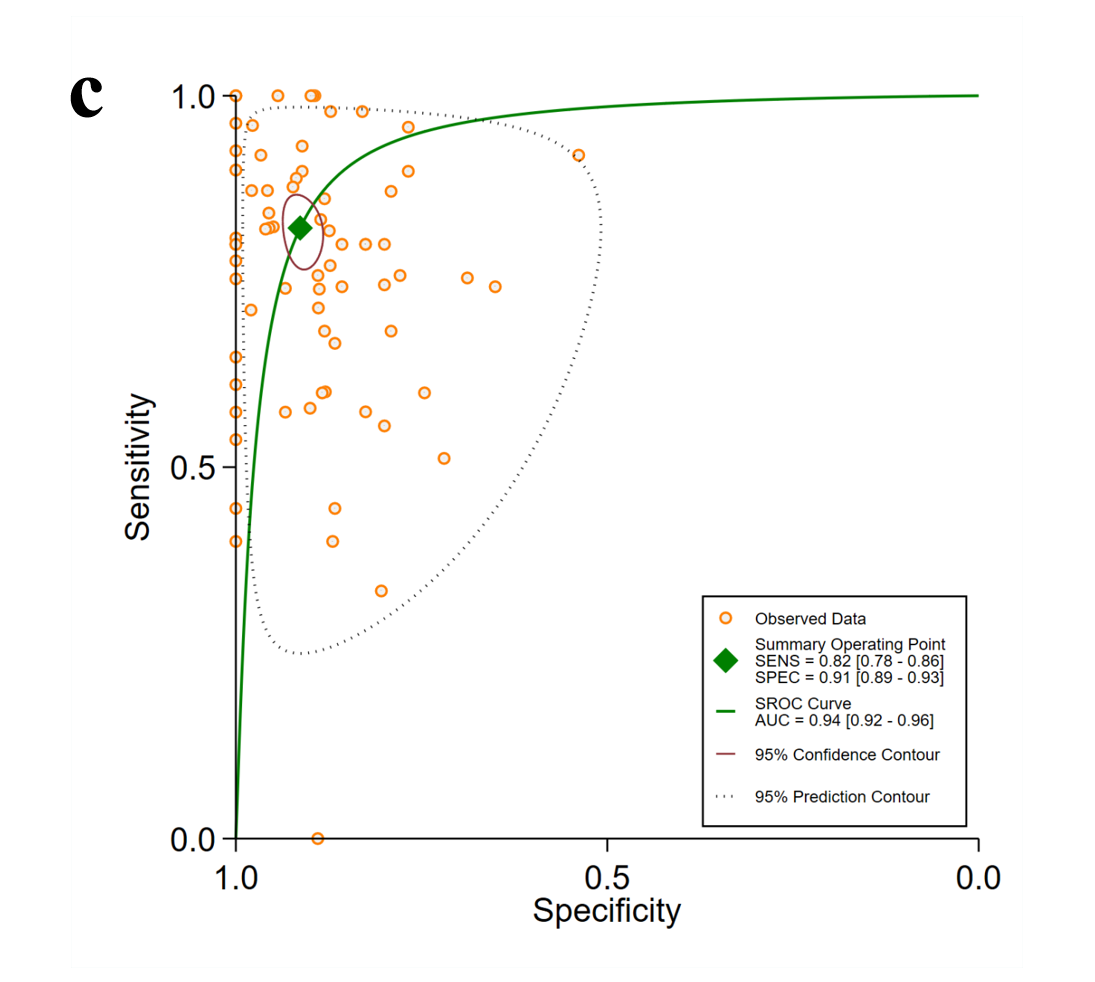

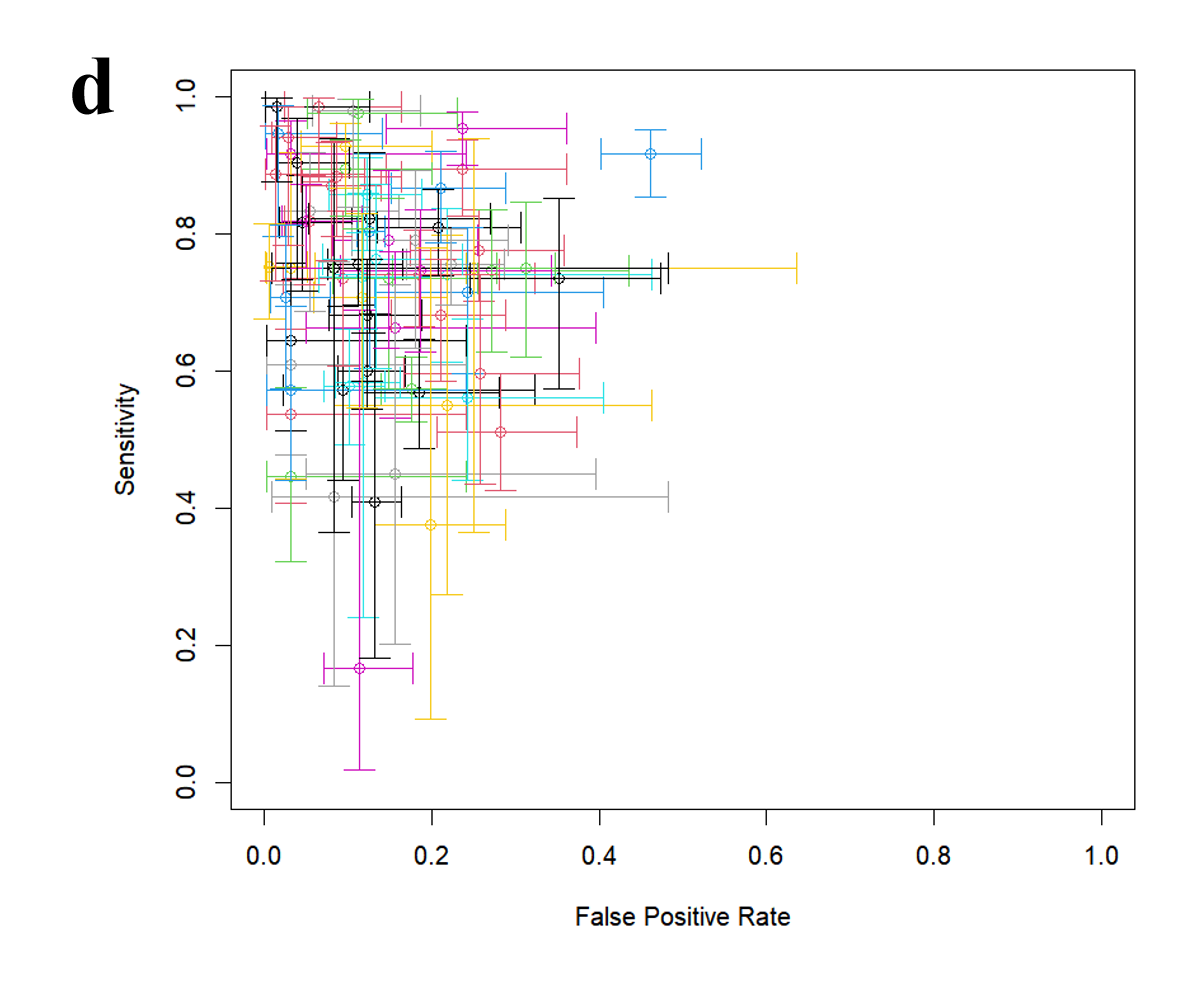


**Figure S11. SROC curve and Cross-hair plot of number of modeling biomarker (> 8, ≤ 8)**

a: SROC curve of number of modeling biomarker > 8 (17 studies with 250 tables)

b: Cross-hair plot of number of modeling biomarker > 8 (17 studies with 250 tables)

c: SROC curve of number of modeling biomarker ≤ 8 (18 studies with 74 tables)

d: Cross-hair plot of number of modeling biomarker ≤ 8 (18 studies with 74 tables)

Abbreviations: SROC=summary receiver operating characteristic; SENS=summary sensitivity; SPEC=summary specificity.

**Figure S12. Forest plot of studies included in the meta-analysis (40 studies)**

**Figure S13 a. Forest plot of artificial intelligence algorithms of machine learning (36 studies with 316 tables)**

**Figure S13 b. Forest plot of artificial intelligence algorithms of deep learning (4 studies with 26 tables)**

**Figure S14 a. Forest plot of studies with external validation (7 studies with 138 tables)**

**Figure S14 b. Forest plot of studies without external validation (33 studies with 204 tables)**

**Figure S15 a. Forest plot of low risk of bias levels (31 studies with 295 tables)**

**Figure S15 b. Forest plot of high risk of bias levels (9 studies with 47 tables)**

**Figure S16 a. Forest plot of year of publication after 2022 (19 studies with 259 tables)**

**Figure S16 b. Forest plot of year of publication before 2022 (21 studies with 83 tables)**

**Figure S17 a. Forest plot of geographical distribution of Asia (18 studies with 243 tables)**

**Figure S17 b. Forest plot of geographical distribution of North America (15 studies with 79 tables)**

**Figure S17 e. Forest plot of geographical distribution of Europe (7 studies with 20 tables)**

**Figure S18 a. Forest plot of sample size > 300 (21 studies with 261 tables)**

**Figure S18 b. Forest plot of sample size ≤ 300 (19 studies with 81 tables)**

**Figure S19 a. Forest plot of blood sample type of serum (27 studies with 117 tables)**

**Figure S19 b. Forest plot of blood sample type of plasma (8 studies with 45 tables)**

**Figure S20 a. Forest plot of biomarker type of protein (25 studies with 119 tables)**

**Figure S20 b. Forest plot of biomarker type of mixed (12 studies with 186 tables)**

**Figure S21 a. Forest plot of number of modeling marker > 8 (17 studies with 250 tables)**

**Figure S21 b. Forest plot of number of modeling marker ≤ 8 (18 studies with 74 tables)**
