# Supplementary material for: AI-Derived Blood Biomarkers for Ovarian Cancer Diagnosis: Systematic Review and Meta-Analysis
Source: J Med Internet Res. 2025 Mar 24;27:e67922. doi: 10.2196/67922 (PMC11976184; doi:10.2196/67922)
Supplement: Multimedia Appendix 6 [file jmir_v27i1e67922_app6.docx]

**Table S1. Participant demographics for the 5 studies without sufficient data**

| **Author, year [ref]** | **Type of analysis** | **Data source (range)** | **Selection criteria** | **Mean or meadian age (Year)** | **N (Number in the dataset)** |
| --- | --- | --- | --- | --- | --- |
| Jing et al., 2023 [77] | Retrospective | Data from Zhujiang Hospital of Southern Medical University, Nanhai District People’s Hospital, Foshan Maternal and Child Health Hospital, Foshan First People’s Hospital, Soochow University Medical Center and Wuxi Maternal and Child Health Hospital | Patients who underwent surgery were pathologically diagnosed with adnexal lesion with complete preoperative serological index data and all blood samples were collected within one week before surgery | NR | 1655(795/342 ^a^) |
| Lu et al., 2022 [78] | Prospective | Data from Sun Yat-Sen Memorial Hospital | NR | NR | 180(NR) |
| Kawakami et al., 2019 [79] | Retrospective | Data from the Department of Obstetrics and Gynecology, the Jikei University School of Medicine (2010-2017) | NR | 52.2 | 435(219/216 ^a^) |
| Song et al., 2018 [80] | Retrospective | Data from Hallym University Chuncheon Sacred Heart Hospital | NR | NR | 193(NR) |
| Elias et al., 2017 [81] | Prospective | Data from the DFCI/BWH Gynecologic Oncology service, and the NECC study (2004–2008) | NR | Training: 56 Testing: 56 | 179(135/44 ^a^) |
| Abbreviation: NR=not reported.  a Training/Testing | | | | | |

**Table S2. Algorithms, type of internal validation, external validation for the 5 studies without sufficient data**

| **Author year [ref]** | **Reference standard** | **Algorithms** | **ML/DL** | **Type of internal validation** | **External validation** |
| --- | --- | --- | --- | --- | --- |
| Jing et al., 2023 [77] | Histopathology | RF, XGBM, SVM, KNN, MLPM | ML | NR | Yes |
| Lu et al., 2022 [78] | NR | Glmnet model | ML | NR | No |
| Kawakami et al., 2019 [79] | NR | GBM, SVM, RF, CRF, NB, NN, EN | ML | Ten-fold cross-validation | No |
| Song et al., 2018 [80] | NR | RF, GA, T-test, LR, LDA, KNN | ML | Five-fold cross-validation | No |
| Elias et al., 2017 [81] | Histopathology | LDA, LR, Multivariate adaptive regression splines, NB, NN, SVM, Functional tree, BaYesian network, Elastic net regression, RF | ML | NR | Yes |
| Abbreviation: CRF: Conditional Random Forest; EN: Elastic Net; GA: Genetic Algorithm; GBM: Gradient Boosting Machine; KNN: K-Nearest Neighbor; LDA: Linear Discriminant Analysis; LR: Logistic Regression; MLPM: Multi-Layer Perceptron Model; NB: Naive BaYes; NN: Neural Network; RF: Random Forest; SVM: Support Vector Machine; XGBM: Extreme Gradient Boosting Model. | | | | | |

**Table S3. Blood sample type, technology, number of biomarkers for the 5 studies without sufficient data**

| **Author year [ref]** | **Blood sample type** | **Technology** | **Biomarker type** | **Number of modeling biomarkers** |
| --- | --- | --- | --- | --- |
| Jing et al., 2023 [77] | Serum | NR | Protein, Mixed | 23 |
| Lu et al., 2022 [78] | Plasma | Nucleic Acids Kit | DNA | 4 |
| Kawakami et al., 2019 [79] | Serum | NR | Protein, Mixed | 32 |
| Song et al., 2018 [80] | Serum | NR | Protein | 4 |
| Elias et al., 2017 [81] | Serum | Immunohistochemistry, RNA isolation kit | RNA | 11 |
| Abbreviation: NR=not reported | | | | |
