# Supplementary figures and images for: AI-Derived Blood Biomarkers for Ovarian Cancer Diagnosis: Systematic Review and Meta-Analysis
Source: J Med Internet Res. 2025 Mar 24;27:e67922. doi: 10.2196/67922 (PMC11976184; doi:10.2196/67922)

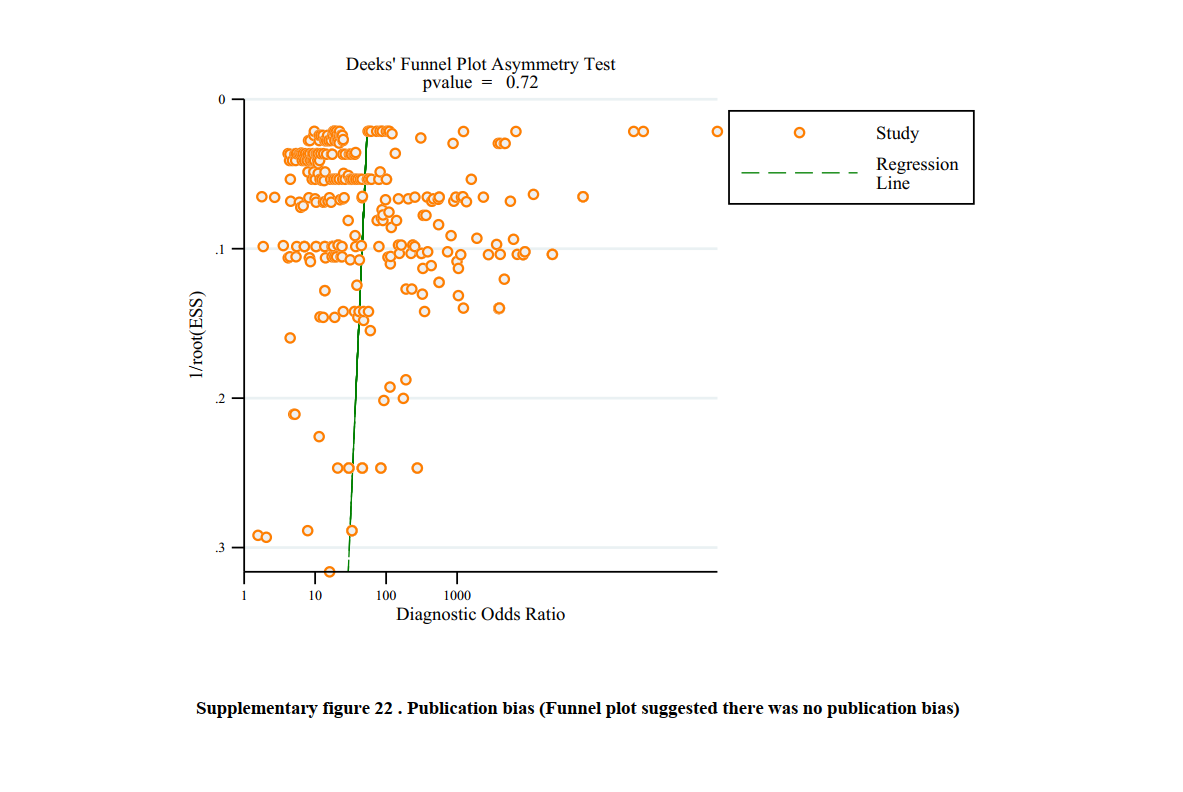


**Multimedia Appendix 7. Publication bias (Funnel plot suggested there was no publication bias)**

Supplement: Multimedia Appendix 7 [file jmir_v27i1e67922_app7.docx]
